# Identification and quantification of the basal and inducible Nrf2-dependent proteomes in mouse liver: Biochemical, pharmacological and toxicological implications

**DOI:** 10.1016/j.jprot.2014.05.007

**Published:** 2014-08-28

**Authors:** Joanne Walsh, Rosalind E. Jenkins, Michael Wong, Adedamola Olayanju, Helen Powell, Ian Copple, Paul M. O’Neill, Christopher E.P. Goldring, Neil R. Kitteringham, B. Kevin Park

**Affiliations:** a MRC Centre for Drug Safety Science, School of Biomedical Sciences, University of Liverpool, Sherrington Buildings, Liverpool, Merseyside L69 3GE, United Kingdom; b AstraZeneca R&D Alderley Park, Safety Assessment UK, Mereside, Alderley Park, Macclesfield, Cheshire SK10 4TG, United Kingdom

**Keywords:** Nrf2, iTRAQ, ENTPD5, CYP2A5, Hepatoproteome, CDDO

## Abstract

The transcription factor Nrf2 is a master regulator of cellular defence: Nrf2 null mice (Nrf2^(−/−)^) are highly susceptible to chemically induced toxicities. We report a comparative iTRAQ-based study in Nrf2^(−/−)^ mice treated with a potent inducer, methyl-2-cyano-3,12-dioxooleana-1,9(11)dien-28-oate (CDDO-me; bardoxolone -methyl), to define both the Nrf2-dependent basal and inducible hepatoproteomes. One thousand five hundred twenty-one proteins were fully quantified (FDR < 1%). One hundred sixty-one were significantly different (*P* < 0.05) between WT and Nrf2^(−/−)^ mice, confirming extensive constitutive regulation by Nrf2. Treatment with CDDO-me (3 mg/kg; i.p.) resulted in significantly altered expression of 43 proteins at 24 h in WT animals. Six proteins were regulated at both basal and inducible levels exhibiting the largest dynamic range of Nrf2 regulation: cytochrome P4502A5 (CYP2A5; 17.2-fold), glutathione-S-transferase-Mu 3 (GSTM3; 6.4-fold), glutathione-S-transferase Mu 1 (GSTM1; 5.9-fold), ectonucleoside-triphosphate diphosphohydrolase (ENTPD5; 4.6-fold), UDP-glucose-6-dehydrogenase (UDPGDH; 4.1-fold) and epoxide hydrolase (EPHX1; 3.0-fold). These proteins, or their products, thus provide a potential source of biomarkers for Nrf2 activity. ENTPD5 is of interest due to its emerging role in AKT signalling and, to our knowledge, this protein has not been previously shown to be Nrf2-dependent. Only two proteins altered by CDDO-me in WT animals were similarly affected in Nrf2^(−/−)^ mice, demonstrating the high degree of selectivity of CDDO-me for the Nrf2:Keap1 signalling pathway.

**Biological significance:**

The Nrf2:Keap1 signalling pathway is attracting considerable interest as a therapeutic target for different disease conditions. For example, CDDO-me (bardoxolone methyl) was investigated in clinical trials for the treatment of acute kidney disease, and dimethyl fumarate, recently approved for reducing relapse rate in multiple sclerosis, is a potent Nrf2 inducer. Such compounds have been suggested to act through multiple mechanisms; therefore, it is important to define the selectivity of Nrf2 inducers to assess the potential for off-target effects that may lead to adverse drug reactions, and to provide biomarkers with which to assess therapeutic efficacy. Whilst there is considerable information on the global action of such inducers at the mRNA level, this is the first study to catalogue the hepatic protein expression profile following acute exposure to CDDO-me in mice. At a dose shown to evoke maximal Nrf2 induction in the liver, CDDO-me appeared highly selective for known Nrf2-regulated proteins. Using the transgenic Nrf2^(−/−)^ mouse model, it could be shown that 97% of proteins induced in wild type mice were associated with a functioning Nrf2 signalling pathway. This analysis allowed us to identify a panel of proteins that were regulated both basally and following Nrf2 induction. Identification of these proteins, which display a large magnitude of variation in their expression, provides a rich source of potential biomarkers for Nrf2 activity for use in experimental animals, and which may be translatable to man to define individual susceptibility to chemical stress, including that associated with drugs, and also to monitor the pharmacological response to Nrf2 inducers.

## Highlights

• Liver proteomes from WT, Nrf2-null and Nrf2-induced mice were compared by iTRAQ
• Of 1521 proteins quantified, 161 were regulated basally and 43 following induction
• Six proteins were both basally and inducibly regulated, with high dynamic ranges
• In order of fold change, these proteins were CYP2A5, GSTM3, GSTM1, ENTPD5, G6PD, EPHX1
• These proteins may yield translatable biomarkers for clinical development

## Graphical abstract

**Figure:**
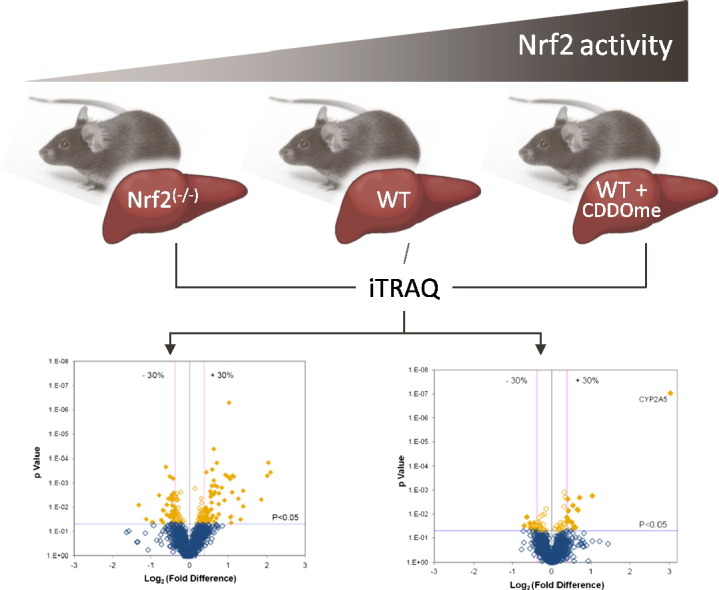

## 1 Introduction

Maintenance of a stable intracellular environment is a prerequisite for normal physiological function. In a manner, somewhat analogous to the immune system, mammalian cells exhibit both innate and adaptive properties that allow them to withstand and respond to a variety of stress stimuli including environmental, dietary and, more recently, pharmaceutical-induced stresses. At the core of this cellular defence strategy is the Keap1:Nrf2 signalling pathway, which regulates expression of a battery of antioxidant proteins and enzymes involved in a variety of mechanisms that function to counter noxious stimuli. In the absence of stress, the transcription factor Nrf2 is retained in the cytoplasm through interaction with its inhibitor protein, Keap1, which targets Nrf2 for ubiquitination and proteasomal degradation. Thus, Nrf2 is rapidly recycled with a half-life of approximately 20 min [1]. Upon exposure to stress stimuli, such as reactive oxygen species and electrophiles, Nrf2 is stabilized and able to translocate to the nucleus where it transactivates target genes that possess an antioxidant responsive element (ARE) in their promoter regions. The precise mechanism through which the Keap1:Nrf2 interaction is disrupted is not fully understood, but the widely accepted ‘hinge and latch’ model [2] envisages that the function of the Keap1 dimer is disrupted by direct modification of sensitive cysteine residues, preventing Nrf2 ubiquitination and blocking access to Keap1 binding sites for newly synthesized Nrf2 molecules. Whilst Keap1-targeted ubiquitination results in highly efficient degradation of Nrf2, it is clear that low levels of Nrf2-mediated signalling do still occur under basal conditions, as evidenced by studies on Nrf2^(−/−)^ mice. Although mice deficient in Nrf2 appear phenotypically normal, examination at the molecular level shows clear differences in gene expression profiles, confirming a constitutive role for Nrf2 in the orchestration of cellular defence [3,4].

In the context of chemical stress, Nrf2^(−/−)^ mice are more vulnerable to the deleterious effects of chemicals toxic to the liver, as well as to several other organs. The animals show enhanced susceptibility to the hepatotoxicity associated with paracetamol [5,6], carbon tetrachloride [7] and ethanol [8], as well as drug-induced injury to the lungs [9,10] and colon [11].

With respect to liver, basal differences between wild type and Nrf2^(−/−)^ mice have been shown both by gene microarray studies and by targeted protein analysis [4,12,13]. More recently, we conducted a global protein expression analysis using iTRAQ-based proteomics and identified two discrete pools of hepatic proteins which display differential expression profiles in wild type and Nrf2^(−/−)^ mice: cytoprotective proteins and proteins involved in lipid metabolism [3]. Pathway analysis confirmed that the cytoprotective proteins found to be down-regulated in Nrf2^(−/−)^ mice were predominantly phase II drug metabolizing enzymes or those involved in the glutathione system. In contrast, proteins involved in lipid metabolism were primarily over-expressed in Nrf2^(−/−)^ mice, indicating an unexpected negative regulation of the fatty acid synthetic pathway.

2-Cyano-3,12-dioxooleana-1,9(11)-dien-28-oic acid (CDDO) was synthesised for its anti-inflammatory properties via the modification of the A and C rings of oleanolic acid [14]. CDDO was found to potently inhibit nitric oxide production, and analogues including CDDO methyl ester (CDDO-me) and the imidazole derivative (CDDO-im) were subsequently synthesised with the aim of optimising potency and bioavailability [15,16]. A link between Nrf2 induction and CDDO treatment was first identified in a study in which the synthetic triterpenoid was shown to potently induce the phase II response in mouse embryonic fibroblasts [17], a response that was abolished in Nrf2-deficient cells. In a later study, CDDO and its derivatives were shown to induce Nrf2 protein levels in vitro along with mRNA levels of the Nrf2 target gene haem oxygenase 1 (*Ho-1*) [18]. Furthermore, the Nrf2 target NAD(P)H dehydrogenase [quinone] 1 (*Nqo1)* was subsequently found to be transcriptionally activated in CDDO-im and CDDO-me treated mice, with induction seen in the liver, lung and small intestine after a single dose [19].

Recently, CDDO-me (under the name bardoxolone methyl) has undergone clinical evaluation for the treatment of chronic kidney disease in diabetic patients [20]. Whilst the therapeutic benefit was promising, the development was terminated in phase III due to a high incidence of adverse reactions [21]. Nevertheless, there remains considerable interest in this class of compounds, particularly in the field of cancer chemotherapy [22]. The precise mechanism by which CDDO and its derivatives mediate their therapeutic effects remains unclear. Preclinical studies have shown that as well as activating the Nrf2 pathway, the compounds also modulate signalling associated with the PPAR-γ receptor [23] and JAK-STAT pathway [24,25] and inhibit both the constitutive and inducible activation of NF-κB [26–28]. Given the side effects identified following repeat administration of CDDO-me to some patients, it is becoming increasingly important to define the effects of CDDO-me at the mRNA and protein level and to ascribe these effects as Nrf2 dependent or independent actions. Furthermore, in order to assess directly the efficacy of CDDO-me and other Nrf2 inducers, biomarkers that specifically reflect both constitutive and induced levels of Nrf2 activity would be invaluable to define the level of human Nrf2 variability and its activation in response to chronic drug exposure. There is consequently a clear imperative to generate a definitive list of Nrf2-regulated genes, since this may yield proteins or protein products that are potential biomarkers for such translational research.

Whilst a comprehensive comparative proteomic characterisation of the liver tissue from Nrf2^(−/−)^ and wild type mice has been conducted at the constitutive level, to date no equivalent analysis of the inducible protein profile has been carried out. For that reason, this study was designed to define the Nrf2-inducible hepatic proteome using the potent Nrf2 activator, CDDO-me. The Nrf2^(−/−)^ mouse provides a useful tool to define which of the changes in protein expression following CDDO-me administration are Nrf2-dependent. When used alongside pharmacological induction of Nrf2, the Nrf2^(−/−)^ model allows the characterisation of the dynamic range of Nrf2 expression and thus allows the identification of candidate biomarkers that might be used to monitor Nrf2 induction in both pre-clinical and translational studies. Through the administration of CDDO-me to Nrf2^(−/−)^ and wild type mice, our aim was to investigate the hepatic proteomic profile of mice treated with CDDO-me and to characterise the differences in protein expression in the absence of Nrf2.

## 2 Materials and methods

### 2.1 Materials

Eight-plex isobaric tags for relative and absolute quantification (iTRAQ) protein labelling kit/reagents were purchased from AB Sciex (Framingham, MA). Sequencing grade trypsin and the ImProm-II Reverse Transcription System were obtained from Promega UK (Southampton, Hants, UK). The RNeasy Mini-kit was purchased from Qiagen (Crawley, UK). The RNA 6000 Nano Kit was from Agilent (Berkshire, UK). ATP citrate lyase (ACL), NQO1 and actin antibodies were from Abcam (Cambridge, UK). CDDO-me was synthesised by Michael Wong (Department of Chemistry, University of Liverpool, UK). All other reagents were of analytical grade and quality and purchased from Sigma (Poole, Dorset, UK).

### 2.2 Animals

All experiments were undertaken in accordance with the Animals (Scientific Procedures) Act 1986, and approved by the University of Liverpool Animal Ethics Committee. Generation of the Nrf2^(−/−)^ mouse and genotyping of progeny have been described elsewhere [29,30]. Non-fasted male littermate wild type and Nrf2^(−/−)^ mice (C57BL6J background) of 10–12 weeks of age were used throughout the study. Mice were housed at 19–23 °C under 12 h light/dark cycles and given free access to food and water.

In order to determine the optimal dose of CDDO-me and a time of induction that resulted in a strong downstream protein response, both dose response and time-course preliminary studies were conducted in wild type mice using NQO1 as a prototypic target protein. For the dose optimisation experiment, mice were administered a single i.p. dose of CDDO-me (0, 0.1, 0.3, 1, 3 or 10 mg/kg in 100 μL DMSO; *n* = 2) at 10 a.m. At 24 h after dosing the animals were culled by exposure to a rising concentration of CO_2_ followed by cardiac puncture. Livers were removed immediately, snap-frozen in liquid N_2_ and stored at − 80 °C. For the time-course assessment, mice were killed at 2, 4, 6 or 24 h after CDDO-me (3 mg/kg; *n* = 4) and the identical subsequent procedure was followed.

For the proteomic study, livers were harvested from wild type and Nrf2^(−/−)^ mice that had been dosed with 3 mg/kg CDDO-me or DMSO vehicle control (*n* = 6), using the protocol as described for the pilot study.

### 2.3 RNA isolation and quality determination

RNA isolation was performed using the RNeasy Mini-kit according to the manufacturer’s instructions, and RNA concentration was determined using a NanoDrop ND-1000 (Labtech, East Sussex, UK). The quality of the RNA was determined using the Agilent RNA 6000 Nano Kit according to the manufacturer’s instructions, with samples analysed using the Agilent 2100 bioanalyzer (Agilent, Berkshire, UK).

### 2.4 cDNA synthesis

cDNA synthesis was carried out using the Promega ImProm-II Reverse Transcription System according to the manufacturer’s instructions, with some minor modifications: 4 μL of RNA at a concentration of 0.5 μg/μL was combined with 1 μL of random primer solution, and nuclease-free dH_2_O was added to give a final volume of 15 μL. The solution was incubated (70 °C; 5 min) and then cooled on ice. A master-mix containing ImProm-II reaction buffer, 6 mM MgCl_2_, dNTP mix and ImProm-II reverse transcriptase in a final volume of 20 μL was added to the RNA solution. Strands were annealed (25 °C; 5 min) and extended (42 °C; 1 h) before the reverse transcriptase was inactivated (70 °C; 15 min). Nuclease-free dH_2_O (160 μL) was added to each tube and cDNA concentration was subsequently determined using the NanoDrop.

### 2.5 Microfluidic cards

Microfluidic cards were designed based on the results of previous proteomic analysis comparing the hepatic profile of basal wild type and Nrf2^(−/−)^ mice [3]. The cards included well established Nrf2-regulated genes, genes encoding proteins identified as Nrf2-regulated in iTRAQ analysis and genes encoding proteins that were not identified by iTRAQ but were associated with pathways identified by MetaCore analysis. Cards were custom made by Applied Biosystems (Paisley, UK). 18S ribosomal RNA was used as a housekeeping gene. Samples were run in a randomised order, as determined using random.org (http://www.random.org/), across 5 TaqMan array cards. A pool of cDNA from all samples was run on each card so that data could be compared across plates.

cDNA was diluted in nuclease-free dH_2_O to a concentration of 2 ng/μL cDNA and analysed according the manufacturer’s instructions using the 7900HT Fast Real-Time PCR System (ABSciex).

### 2.6 Microfluidic data analysis

Data was analysed using the comparative C_T_ method (ΔΔC_T_). C_T_ values were determined using the RQ manager 1.2 component of the 7900HT Fast System software. The threshold was manually set to a value of 0.3 for all plates. Gene expression was quantified relative to the sample pool run on the same plate and normalised to 18S gene expression. Relative expression of genes was compared in wild type vehicle treated and CDDO-me treated mice, vehicle treated wild type and Nrf2^(−/−)^ mice and Nrf2^(−/−)^ vehicle treated and CDDO-me treated mice by two way ANOVA with Tukey multiple comparison testing. Statistical analysis was performed using StatsDirect version 2.7.9 (StatsDirect Ltd, Altrincham, UK).

### 2.7 iTRAQ labelling and mass spectrometric analysis of liver homogenates

Liver samples (~ 100 mg wet weight) were dounce homogenised in 0.5 M triethylammonium bicarbonate/0.1% SDS and subjected to a freeze–thaw cycle (− 80 °C for 30 min), before sonication (3 x 10 s at 5 μm amplitude) and centrifugation at 17,000*g* for 10 min at 4 °C. Aliquots of each sample (75 μg protein [31]) were denatured and reduced and sulphydryl groups were capped with MMTS according to the manufacturer’s 8-plex protocol (Applied Biosystems, CA). The samples were digested with trypsin overnight, labelled with iTRAQ isobaric tags (113–121) and mixed in equal proportions. Unbound reagent and trypsin were removed by cation exchange chromatography. Fractions were desalted using a macroporous C_18_ column (Agilent, Santa Clara, CA) on a Vision workstation and dried by centrifugation under vacuum (SpeedVac, Eppendorf). Samples were analysed on a Triple TOF 5600 mass spectrometer (AB Sciex) and were delivered into the instrument by automated in-line liquid chromatography Eksigent NanoUltra cHiPLC System mounted with microfluidic trap and analytical column (15 cm × 75 μm) packed with ChromXP C_18_-CL 3 μm via a nano-electrospray source head and 10 μm inner diameter PicoTip (New Objective, MA). The precolumn was washed for 10 min at 2 μL/min with 2% ACN/0.1% FA. A gradient from 2% ACN/0.1% FA (v/v) to 50% I/0.1% FA (v/v) in 90 min was applied at a flow rate of 300 nL/min.

The MS was operated in positive ion mode with survey scans of 250 ms, and with an MS/MS accumulation time of 100 ms for the 25 most intense ions (total cycle time 2.5 s). A threshold for triggering of MS/MS of 100 counts per second was used, together with dynamic exclusion for 12 s and rolling collision energy, adjusted for the use of iTRAQ reagent in the Analyst method. Information-dependent acquisition was powered by Analyst TF 1.5.1 software, using mass ranges of 400–1600 amu in MS and 100–1400 amu in MS/MS. The instrument was automatically calibrated after every fifth sample using a beta-galactosidase digest.

### 2.8 iTRAQ protein identification and statistical analyses

Liver samples from wild type and Nrf2^(−/−)^ mice, treated with CDDO-me or DMSO vehicle control, were analysed across four iTRAQ runs with a comparator pooled sample incorporated in each run for normalisation between iTRAQ experiments. Samples (*n* = 6) for each treatment were randomised across the four runs to minimise label bias. Ratios for each iTRAQ label were obtained, using the common pool as the denominator (iTRAQ label 113). Data analysis was performed using ProteinPilot software (version 3, Applied Biosystems, Warrington, UK). The data were analysed with MMTS as a fixed modification of cysteine and biological modifications. The SwissProt database was searched with a confidence interval of 95% and also screened in reverse to facilitate false discovery rate (FDR) analysis. Proteins identified from peptides with more than 95% confidence and a global FDR of less than 1% were included in the statistical analysis.

The *limma* package within the R programming environment [32] allowed simultaneous comparisons between multiple treatments using design and contrast matrices. This open source software generates a linear regression model (lm) to facilitate the analysis of differential protein expression. Mean fold changes were calculated and analysis was conducted on the logged fold-change values. Unadjusted (raw) *P* values and *P* values following Benjamini–Hochberg (BH) correction for multiple testing were determined.

Nrf2- and CDDO-me-dependent protein expression was defined by comparing Nrf2^(−/−)^ DMSO with wild type DMSO (group A), wild type DMSO with wild type CDDO-me (group B) and Nrf2^(−/−)^ DMSO with Nrf2^(−/−)^ CDDO-me mice (group C). The resulting protein lists for genetic disruption and pharmacological pathway activation were compared to identify changes that were both *common* and *unique* to Nrf2 and CDDO-me in a similar manner to the gene expression studies performed in Keap1^(−/−)^ and triterpenoid treated mice reported by Yates et al. [33].

### 2.9 Ontology and pathway analysis

Pathway analysis was performed as previously described using MetaCore from GeneGo Inc [3]. The software was used in order to identify the pathways most significantly differentially regulated in livers of wild type and Nrf2^(−/−)^ mice as well as in wild type vehicle control and wild type CDDO-me treated animals.

### 2.10 Immunoblotting for Nrf2 target proteins

In order to confirm the iTRAQ-identified expression changes in key Nrf2- and CDDO-driven gene targets, western immunoblotting was undertaken for NQO1, ACL, CYP2A5 and ENTPD5 using methods as described previously [3]. A polyclonal goat anti-NQO1 antibody (ab2346, Abcam plc, Cambridge, UK) was used at a dilution of 1:5000, while a monoclonal rabbit anti-ENTPD5 antibody (ab108603, Abcam plc) was used at 1:10,000. The chicken anti-CYP2A5 antibody was generously provided by Risto Juvonen, University of Eastern Finland, Kuopio, Finland.

### 2.11 Enhancer element binding site analysis

Proteins that were found to be regulated by Nrf2 at both the constitutive and inducible levels were subjected to enhancer element binding site analysis using the Genomatix software suite (v3.1). Full length gene and promoter (3000 bp) DNA sequences were interrogated for consensus Nrf2 binding sites using the MatInspector search [34] within the MAF and AP1 related factor subgroup (V$AP1R). Full length gene sequences were retrieved from Entrez gene and promoter sequences were extracted using the Genomatix Gene2Promoter tool. Matrix similarity was optimised and the core similarity threshold was set to 0.75.

## 3 Results

### 3.1 Induction of Nrf2 by CDDO-me

**Fig. 1:**
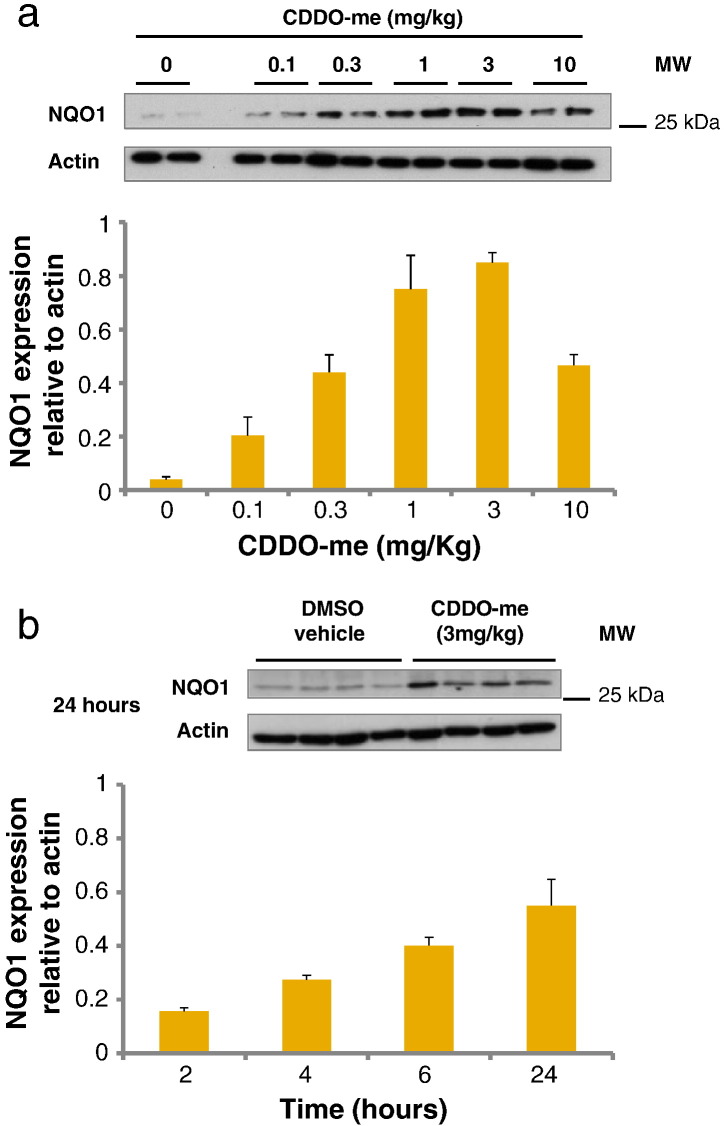
Immunoblots of liver homogenates from pilot study wild type mice treated with CDDO-me in DMSO (i.p.). Immunoblots for NQO1 were used in order to determine the dose (a) and duration (b) of CDDO-me treatment that results in maximum NQO1 induction. Densitometric analysis of the immunoblots shows NQO1 expressed relative to actin. Error bars represent SEM (*n* = 2, a; *n* = 4, b).

Preliminary studies were performed in order to determine a dose of CDDO-me and a suitable timepoint that would enable analysis of downstream protein expression in the liver resulting from Nrf2 induction after a single administration. The dose range used was based on a study in ICR mice [19]. Nrf2 induction was determined by NQO1 western immunoblotting (Fig. 1). The 24 h timepoint and a dose of 3 mg/kg CDDO-me were found to produce the highest NQO1 signal, with the response diminishing at higher doses. Consequently 24 h exposure to 3 mg/kg CDDO-me was selected for future use.

**Fig. 2:**
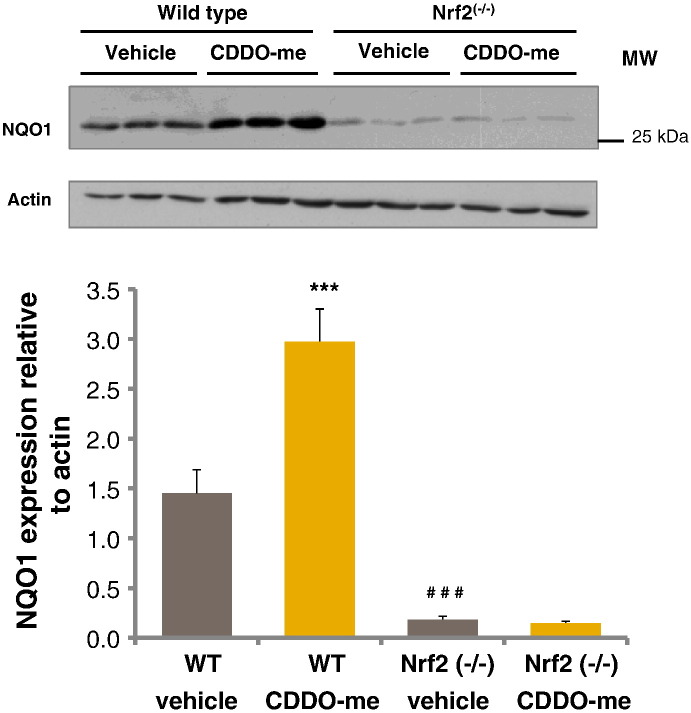
Immunoblots of liver homogenates from wild type and Nrf2^(−/−)^ mice treated with CDDO-me in DMSO (i.p.) and culled 24 h later. Densitometric analysis of the immunoblots shows NQO1 expressed relative to actin. Error bars represent SEM (*n* = 6). Statistical analysis was performed using a two way ANOVA with Tukey multiple comparison testing. NQO1 expression was compared in wild type vehicle and CDDO-me treated mice (***P < 0.001), vehicle treated wild type and Nrf2^(−/−)^ mice (^###^*P* < 0.001), and Nrf2^(−/−)^ vehicle and CDDO-me treated mice (no statistical difference).

Induction of Nrf2 at this dose in the subsequent study was confirmed by analysis of NQO1. Fig. 2 shows a representative blot of NQO1 levels in each treatment group and densitometric analysis of expression of NQO1 in all animals in the study (*n* = 6). Administration of CDDO-me resulted in a two-fold increase in NQO1 in wild type animals at 24 h but no change in the Nrf2^(−/−)^ mice. NQO1 was expressed at a level that was 8-fold lower in Nrf2^(−/−)^ control animals when compared to their wild type counterparts.

### 3.2 Microfludic TaqMan low density array (TLDA) cards

Microfluidic TLDA cards were custom-designed. Each card allows the simultaneous amplification of 48 gene targets in 8 samples. Target genes were selected on the basis of our previous proteome comparison of Nrf2^(−/−)^ and wild type mice. These were either directly identified as Nrf2-regulated or were shown by MetaCore analysis to reside in Nrf2-regulated pathways [3].

**Fig. 3:**
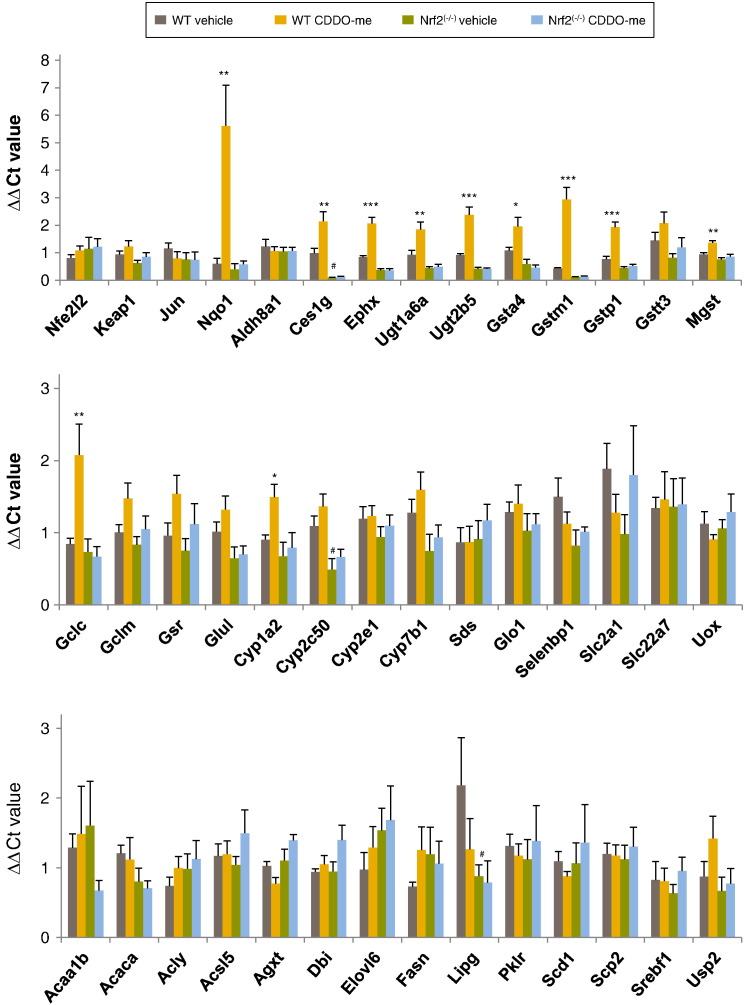
Relative expression of mRNA in livers of wild type and Nrf2^(−/−)^ mice treated with CDDO-me or DMSO vehicle control, as detected by Microfluidic TaqMan low density array analysis. Levels of mRNA for NQO1, CES1G, EPHX, UGT1A6A, UGT2B5, GSTA4, GSTM1, GSTP1, MGST, GCLC and CYP1A2 were statistically significantly higher in CDDO-me treated wild type animals when compared to vehicle control (*), while CES1G, CYP2C50 and LIPG were statistically significantly lower in Nrf2^(−/−)^ vehicle control animals when compared to their wild type counterparts (^#^). There was no statistical difference in mRNA levels of CDDO-me and vehicle control treated Nrf2^(−/−)^ animals. Statistical significance was assessed using a two way ANOVA with Tukey multiple comparison testing.

cDNA reverse transcribed from RNA extracted from the livers of vehicle control and CDDO-me treated wild type and Nrf2^(−/−)^ mice (*n* = 8) was amplified using real-time PCR, with data analysed using the ΔΔC_T_ method. Pooled cDNA from all samples was included on each plate, and expression of all other samples on the plate was expressed relative to the pool and normalised to expression of the housekeeping gene 18S rRNA. Statistical analysis was conducted on all genes for which complete data sets were obtained in > 4 of the 8 samples. On this basis, five of the genes, *Abcc1*, *Abcc4*, *Bhmt*, *Fabp5* and *Prdx6*, were excluded. The mean relative expression of each gene was calculated and standard error of the mean was determined (Fig. 3).

The expression of genes was compared in wild type vehicle control treated and wild type CDDO-me treated mice, wild type and Nrf2^(−/−)^ vehicle control treated mice and Nrf2^(−/−)^ vehicle control and Nrf2^(−/−)^ CDDO-me treated mice (two way ANOVA with Tukey multiple comparison testing). Eleven genes, *Gstm1*, *Ephx*, *Ugt2b5*, *Gstp1*, *Nqo1*, *Mgst*, *Ces1g*, *Cyp1a2*, *Gsta4*, *Ugt1a6a* and *Gclc*, were expressed at a significantly higher level in wild type CDDO-me treated animals when compared to the vehicle control group, while three genes, *Ces1g, Cyp2c50* and *Lipg*, were expressed at a significantly lower level in the Nrf2^(−/−)^ control treated mice when compared to their wild type counterparts. None of the genes were significantly differentially expressed when the Nrf2^(−/−)^ vehicle control and Nrf2^(−/−)^ CDDO-me treated groups were compared. The genes that were up-regulated with CDDO-me treatment in wild type animal are associated with drug metabolism and their regulation by Nrf2 has been well characterised. However, expression of the lipid metabolism genes that were included in the TLDA analysis was not significantly altered with CDDO-me treatment.

Nrf2 mRNA was expressed in the Nrf2^(−/−)^ animals; however, this is consistent with the molecular lesion introduced, in which exon 5 of the Nrf2 gene is absent, rendering the Nrf2 protein non-functional. This is in line with results of previous studies [35].

### 3.3 Characterization of the constitutive Nrf2-responsive hepatic proteome

A comparative iTRAQ-based proteomic analysis of livers from Nrf2^(−/−)^ and wild type mice was conducted. In order to define Nrf2-dependent expression of proteins at both the basal and inducible (24 h post dosing) levels, proteins were extracted from both DMSO vehicle treated mouse livers and those treated with CDDO-me (3 mg/kg). Proteome profiling of all mouse liver samples yielded 3655 unique identifications at an FDR of < 1%. From this total, 1521 were shown to be quantifiable in at least four mice belonging to each of the four treatment groups, and these proteins were incorporated in the full statistical analysis. Table 1 includes the list of 87 proteins that were up- or down-regulated by at least 30% (*P* < 0.05) in Nrf2^(−/−)^ mice when compared to wild type animals at the basal level. By applying a relatively non-stringent statistical analysis (without correction for multiple testing), a total of 161 liver proteins were deemed statistically different between wild type and Nrf2^(−/−)^ mice (irrespective of the fold change), and are detailed in Supplementary Table 1. Whilst this level of statistical analysis is insufficient for unequivocal designation of Nrf2-driven proteins, it yields a sufficient number of nominally Nrf2-regulated proteins to provide candidates for biomarker assessment and to allow meaningful ontology and pathway analysis. As noted by Subramanian et al. [36], the application of stringent multiple testing correction algorithms (such as Bonferroni or Benjamini Hochberg analyses) to large scale global analysis data can preclude the identification of modest expression changes that can collectively modulate a specific pathway. Of the 161 Nrf2-regulated proteins identified, 94 were expressed at a lower level in the Nrf2^(−/−)^ mice and 67 were up-regulated. This is in line with our previous study, and with genomic studies, which show both positive and negative regulation through the Nrf2 transcription pathway. Protein expression differences between Nrf2^(−/−)^ and wild type animals were evaluated to identify the primary biological functions and pathways associated with these genes. Analysis using MetaCore identified 48 pathways that were significantly differentially regulated in the livers of wild type and Nrf2^(−/−)^ mice (Table 2; *P* < 0.05).

A comparative iTRAQ-based proteomic analysis of livers from Nrf2^(−/−)^ and wild type mice was conducted. In order to define Nrf2-dependent expression of proteins at both the basal and inducible (24 h post dosing) levels, proteins were extracted from both DMSO vehicle treated mouse livers and those treated with CDDO-me (3 mg/kg). Proteome profiling of all mouse liver samples yielded 3655 unique identifications at an FDR of < 1%. From this total, 1521 were shown to be quantifiable in at least four mice belonging to each of the four treatment groups, and these proteins were incorporated in the full statistical analysis. Table 1 includes the list of 87 proteins that were up- or down-regulated by at least 30% (*P* < 0.05) in Nrf2^(−/−)^ mice when compared to wild type animals at the basal level. By applying a relatively non-stringent statistical analysis (without correction for multiple testing), a total of 161 liver proteins were deemed statistically different between wild type and Nrf2^(−/−)^ mice (irrespective of the fold change), and are detailed in Supplementary Table 1. Whilst this level of statistical analysis is insufficient for unequivocal designation of Nrf2-driven proteins, it yields a sufficient number of nominally Nrf2-regulated proteins to provide candidates for biomarker assessment and to allow meaningful ontology and pathway analysis. As noted by Subramanian et al. [36], the application of stringent multiple testing correction algorithms (such as Bonferroni or Benjamini Hochberg analyses) to large scale global analysis data can preclude the identification of modest expression changes that can collectively modulate a specific pathway. Of the 161 Nrf2-regulated proteins identified, 94 were expressed at a lower level in the Nrf2^(−/−)^ mice and 67 were up-regulated. This is in line with our previous study, and with genomic studies, which show both positive and negative regulation through the Nrf2 transcription pathway. Protein expression differences between Nrf2^(−/−)^ and wild type animals were evaluated to identify the primary biological functions and pathways associated with these genes. Analysis using MetaCore identified 48 pathways that were significantly differentially regulated in the livers of wild type and Nrf2^(−/−)^ mice (Table 2; *P* < 0.05).

### 3.4 Characterization of the CDDO-me inducible Nrf2-dependent hepatic proteome

**Fig. 4:**
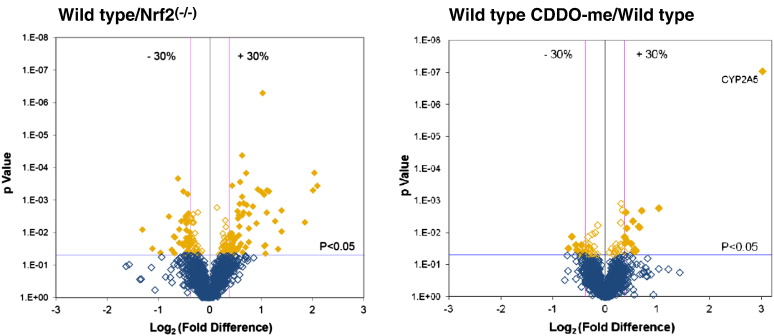
Volcano plots of the proteins quantified during iTRAQ analysis comparing (a) wild type vehicle control and Nrf2^(−/−)^ vehicle control and (b) wild type CDDO-me and wild type vehicle control mice. Each point represents the difference in expression (fold-change) between the two groups of mice plotted against the level of statistical significance. Dotted vertical lines represent differential expression differences of ± 30%, while the dotted horizontal line represents a significance level of *P* < 0.05. Proteins represented by a filled yellow square are those with expression that differs by at least 30% at a statistically significant level.

Following administration of CDDO-me, 43 proteins were either up- or down-regulated in wild type mice. Of these, only 2 were similarly altered in Nrf2^(−/−)^ mice. Complete lists of proteins whose expression was altered by CDDO-me in wild type and Nrf2^(−/−)^ mice are provided in Supplementary Tables 2 and 3, respectively. These data are displayed graphically in Fig. 4, which presents the fold difference for each individual protein identified in at least 4 mice (1521 in total) plotted against the *P* value; Fig. 4A represents the comparison between wild type and Nrf2^(−/−)^ mice at the basal level, whilst the effect of CDDO-me treatment in wild type animals is shown in Fig. 4B. Inspection of these plots suggests that the influence of Nrf2 upon the basal proteome may be generally more profound than the effect of acute induction. Overall, more proteins lie above the statistical cut-off of *P* < 0.05 with the comparison at the basal level than are statistically induced by CDDO-me. Moreover, with the exception of CYP2A5 (labelled in Fig. 4B), the fold differences between wild type and Nrf2^(−/−)^ mice at the constitutive level comprised a far greater range than those following CDDO-me treatment.

Following administration of CDDO-me, 43 proteins were either up- or down-regulated in wild type mice. Of these, only 2 were similarly altered in Nrf2^(−/−)^ mice. Complete lists of proteins whose expression was altered by CDDO-me in wild type and Nrf2^(−/−)^ mice are provided in Supplementary Tables 2 and 3, respectively. These data are displayed graphically in Fig. 4, which presents the fold difference for each individual protein identified in at least 4 mice (1521 in total) plotted against the *P* value; Fig. 4A represents the comparison between wild type and Nrf2^(−/−)^ mice at the basal level, whilst the effect of CDDO-me treatment in wild type animals is shown in Fig. 4B. Inspection of these plots suggests that the influence of Nrf2 upon the basal proteome may be generally more profound than the effect of acute induction. Overall, more proteins lie above the statistical cut-off of *P* < 0.05 with the comparison at the basal level than are statistically induced by CDDO-me. Moreover, with the exception of CYP2A5 (labelled in Fig. 4B), the fold differences between wild type and Nrf2^(−/−)^ mice at the constitutive level comprised a far greater range than those following CDDO-me treatment.

It is also notable that a sizable proportion of proteins were expressed at a lower level in the wild type animals than in the Nrf2^(−/−)^ animals, indicating a level of negative regulation by Nrf2. In contrast, the majority of the changes observed following CDDO-treatment were up-regulations. The proteins up- or down-regulated by at least 30% in wild type and Nrf2^(−/−)^ mice are included in Table 1. In wild type animals, 18 proteins were induced compared with just 4 whose expression was decreased after CDDO-me. Of the 18 protein induced, 16 were uniquely up-regulated in wild type but not in Nrf2^(−/−)^ animals. As with the constitutively regulated proteins, proteins induced by CDDO-me were heavily dominated by drug metabolizing enzymes and proteins involved in lipid synthesis/metabolism. Notably, however, there was no indication that CDDO-me resulted in a reduced expression of proteins involved in fatty acid synthesis. A negative regulation of such proteins, including ACL, fatty acid synthase and acyl coA desaturase, at the *constitutive* level was observed both in the current iTRAQ analysis and in our previous investigation [3]. A similar effect has been shown at the mRNA level by Tanaka et al. [37]. CDDO-im has also been shown by other groups to cause down-regulation of genes involved in the synthesis of fatty acid at the mRNA level in wild type mice [33,38] but this was not confirmed at the mRNA or protein level in our study with CDDO-me. Several of the key lipid metabolic enzymes showed a numerically reduced expression following CDDO-me, such as ACL, which showed a 25% reduction following induction. These values were not statistically significant.

Analysis using MetaCore identified 8 pathways that were significantly altered in the livers of wild type mice treated with CDDO-me, when compared to vehicle control treated mice (Table 3; *P* < 0.05).

### 3.5 Characterisation of proteins regulated by Nrf2 at both basal and CDDO-me inducible level

Six proteins were basally expressed at a significantly lower level in Nrf2^(−/−)^ when compared to wild type and were also significantly up-regulated following CDDO-me treatment in wild type mice, with expression differences in each case of > 30%. A summary of the function of the proteins is given in Table 4. Of the proteins identified as most significantly regulated by Nrf2, GSTM3, GSTM1 and EPXH1 are well characterised as Nrf2-regulated proteins. The regulation of CYP2A5 and UDPGDH by Nrf2 has also been noted previously [4,39]. However, as far as we are aware, Nrf2 regulation of ENTPD5 at the protein level is a novel finding of this study.

### 3.6 Western immunoblotting validation of regulation of CYP2A5 and ENTPD5 by Nrf2

**Fig. 5:**
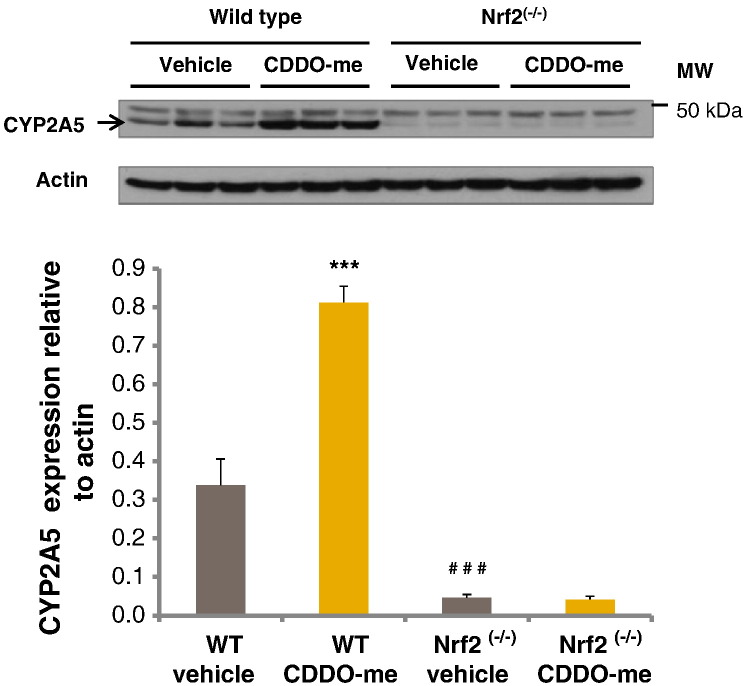
CYP2A5 immunoblots of liver homogenates from wild type and Nrf2^(−/−)^ mice treated with CDDO-me or DMSO vehicle control (i.p.) and culled 24 h later. Densitometric analysis of the immunoblots shows CYP2A5 expressed relative to actin. Error bars represent SEM (*n* = 6). Statistical analysis was performed using a two way ANOVA with Tukey multiple comparison testing. CYP2A5 expression was compared in wild type vehicle and CDDO-me treated mice (***P < 0.001), vehicle treated wild type and Nrf2^(−/−)^ mice (^###^*P* < 0.001), and Nrf2^(−/−)^ vehicle and CDDO-me treated mice (no statistical difference).

**Fig. 6:**
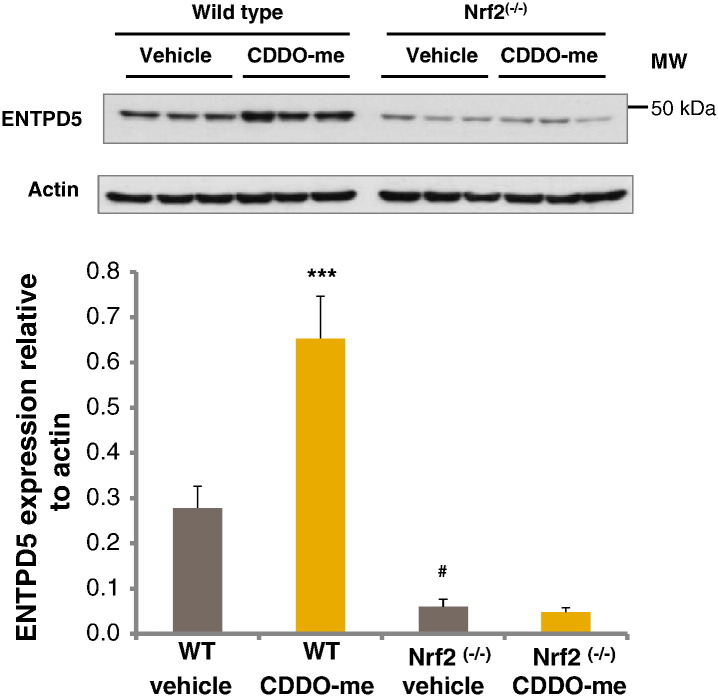
ENTPD5 immunoblots of liver homogenates from wild type and Nrf2^(−/−)^ mice treated with CDDO-me or DMSO vehicle control (i.p.) and culled 24 h later. Densitometric analysis of the immunoblots shows ENTPD5 expressed relative to actin. Error bars represent SEM (*n* = 6). Statistical analysis was performed using a two way ANOVA with Tukey multiple comparison testing. ENTPD5 expression was compared in wild type vehicle and CDDO-me treated mice (***P < 0.001), vehicle treated wild type and Nrf2^(−/−)^ mice (^#^*P* < 0.05), and Nrf2^(−/−)^ vehicle and CDDO-me treated mice (no statistical difference).

Western immunoblotting was performed in order to validate the differences noted in expression of CYP2A5 and ENTPD5 (Figs. 5 and 6 respectively). Densitometric analysis of immunoblots identified a 2.4-fold induction in CYP2A5 levels in wild type mice treated with CDDO-me when compared to vehicle control mice, while no induction was identified in Nrf2^(−/−)^ mice treated with the triterpenoid. Expression of CYP2A5 was 7.4-fold lower in vehicle control Nrf2^(−/−)^ animals when compared to their wild type counterparts. ENTPD5 expression was induced 2.3-fold in CDDO-me treated wild type animals, with no induction in Nrf2^(−/−)^ mice. Furthermore, comparison of the vehicle control groups showed that ENTPD5 expression was reduced by 4.6-fold in Nrf2^(−/−)^ animals.

### 3.7 ACL in CDDO-me treated mice

**Fig. 7:**
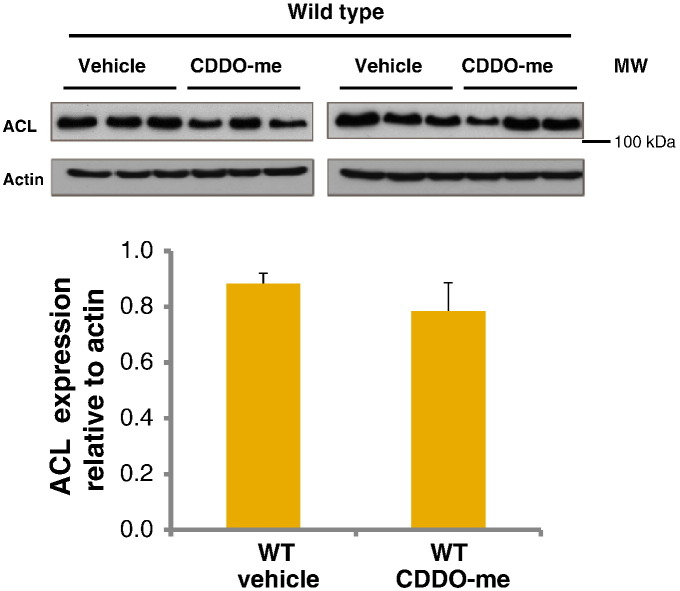
ACL immunoblots of liver homogenates from wild type mice treated with CDDO-me or DMSO vehicle control (i.p.) and culled 24 h later. Densitometric analysis of the immunoblots shows ACL expressed relative to actin. Error bars represent SEM (*n* = 6). Statistical analysis was performed using a Student’s *t*-test; there was no statistical difference between the two groups.

In order to further investigate potential differences in fatty acid metabolism enzymes in vehicle control and CDDO-me treated wild type mice, a western immunoblot for ACL was performed (Fig. 7). The results confirmed the iTRAQ analysis showing that there was no statistical difference in expression of the protein between wild type animals in the vehicle control and those treated with CDDO-me.

### 3.8 Enhancer element binding site analysis of the proteins regulated by Nrf2 at both the constitutive and inducible levels

The Genomatix software suite was used in order to interrogate full length gene and promoter (3000 bp) DNA sequences from proteins identified as regulated by Nrf2 at both the constitutive and inducible levels for consensus Nrf2 binding sites. Results presented in Supplementary Table 4 focus on three transcription factor binding sites within the MAF and AP1 related factor subgroup: NF-E2 p45, antioxidant response elements and binding sites for heterodimers with small Maf-proteins. Thirty-two consensus binding sites were identified in the *Entpd5* gene (8 in the promoter region and a further 24 in the full length sequence), 13 in the *Cyp2a5* gene (2 in the promoter region, 11 in the full length sequence), 9 in the *Gstm3* gene (7 in the promoter region and 2 in the full length gene), 2 in the *Gstm1* gene (2 in the full length gene), 34 in the *Ephx1* gene (11 in the promoter and 23 in the full length gene) and 19 in the *Ugdh* gene (12 in the promoter and 7 in the full length gene).

The Genomatix software suite was used in order to interrogate full length gene and promoter (3000 bp) DNA sequences from proteins identified as regulated by Nrf2 at both the constitutive and inducible levels for consensus Nrf2 binding sites. Results presented in Supplementary Table 4 focus on three transcription factor binding sites within the MAF and AP1 related factor subgroup: NF-E2 p45, antioxidant response elements and binding sites for heterodimers with small Maf-proteins. Thirty-two consensus binding sites were identified in the *Entpd5* gene (8 in the promoter region and a further 24 in the full length sequence), 13 in the *Cyp2a5* gene (2 in the promoter region, 11 in the full length sequence), 9 in the *Gstm3* gene (7 in the promoter region and 2 in the full length gene), 2 in the *Gstm1* gene (2 in the full length gene), 34 in the *Ephx1* gene (11 in the promoter and 23 in the full length gene) and 19 in the *Ugdh* gene (12 in the promoter and 7 in the full length gene).

## 4 Discussion

Loss of Nrf2 signalling in the Nrf2^(−/−)^ mouse model has been shown to increase susceptibility to various forms of chemical-induced pathologies, including hepatotoxicity associated with acetaminophen [5,6] and carbon tetrachloride [7], lung damage induced by butylated hydroxyl toluene [40] and lipopolysaccharide-induced sepsis [41]. These studies involved acute administration of single doses of the chemical toxins, suggesting that the enhanced susceptibility in the Nrf2^(−/−)^ animals was due to lower basal expression of cellular defence proteins, rather than an abrogated ability to respond to the treatment by up-regulation of the Nrf2-driven genes, since it is unlikely that such an adaptive response could occur within the time-frame of the acute toxicity. This notion is supported by our recent proteomic study of acetaminophen (APAP) hepatotoxicity, which showed very few APAP-induced changes at the protein level within the timeframe of the toxic response (unpublished data). In other cases, such as the neurotoxicity seen with MPTP, gastrointestinal toxicity with dextran-sulphate and stomach neoplasias induced by benzopyrene, Nrf2 was shown to protect against chronic administration of the toxins and this may reflect reduced induction of a protective response in the Nrf2^(−/−)^ animals. Thus, both lower constitutive levels of Nrf2 regulated proteins, as well as a reduced ability to up-regulate these proteins are likely to contribute to the enhanced susceptibility of Nrf2^(−/−)^ mice to chemical stress. It is thus important to know whether the same proteins are regulated at both constitutive and inducible levels, or whether different populations of cellular defence proteins may be involved in the acute and chronic protection afforded by Nrf2 in these different animal models. It is also important to define the dynamic range of expression of Nrf2 proteins and how this might impact upon the toxicity of specific chemicals and influence the susceptibility of particular species and individuals in the human population.

In our previous study [3] we compared the basal liver proteomes of Nrf2^(−/−)^ and wild type mice to identify proteins involved in cellular defence against acute chemical insults; however, no similar study has yet been carried out at the protein level in an Nrf2-induced mouse model. Here, we have extended our analysis to define the Nrf2-inducible protein population using the most potent activator of Nrf2 currently available, CDDO-me. CDDO-me was recently investigated in man as a potential therapeutic agent for the treatment of type 2 diabetic kidney disease [42]. Other Nrf2 inducers are now being developed for a range of therapeutic indications. For example, dimethyl fumarate has recently been approved by the FDA as a treatment for reducing the incidence of relapse in multiple schlerosis patients [43]. Thus knowledge of the pattern of protein induction becomes essential in order to predict the biochemical, pharmacological and toxicological consequences of sustained Nrf2 activation.

Overall, out of 1556 proteins identified and quantified, 161 proteins were different at the basal level between wild type and Nrf2^(−/−)^ mice, whereas only 43 were similarly altered following CDDO-me treatment of wild type animals. What was particularly striking, however, was the lack of overlap between these two lists of proteins: only 6 proteins were both lower in the Nrf2^(−/−)^ mice and induced by CDDO-me in the wild types. These were CYP2A5, GSTM3, GSTM1, ENTPD5, UDPGDH and EPHX1. Superficially, this lack of concordance between the basal and inducible protein populations suggests that two discrete subsets of Nrf2 target proteins exist, one that responds to a loss of Nrf2 and one that is up-regulated following chemical activation of the Nrf2 signalling pathway, with only a limited overlap between the two. Whilst this concept has a plausible toxicological rationale, in that a cell’s constitutive defence system must be wide-ranging and able to counter a broad range of chemical insults, whereas an inducible response can be tailored to the specific toxin to which the cell is exposed, such an interpretation of these proteomic data must be viewed with caution. Several differences exist between the two methods used to modulate Nrf2 activity, which could, either directly or indirectly, alter the protein expression profiles at the basal and inducible levels. First, the greater abundance of constitutively regulated proteins may reflect longer term or compensatory changes in the knockout animals, which would not be apparent following a single, acute treatment with an inducing agent. Furthermore, although the 24 h post-CDDO-me timepoint was optimum for NQO1 protein induction, this may not be the case for all target proteins and thus a single “snapshot” of the inducible proteome may not capture the entirety of up-regulated proteins. Alternatively, it is possible that CDDO-me causes Nrf2 activation in cell types other than hepatocytes, for example, Kupffer or stellate cells, both of which possess inducible Nrf2 pathways [44,45], and that the observed protein changes from CDDO-me treatment could result from secondary cell-to-cell signalling events. These alternative explanations require further investigation before concluding that the difference between the two proteomes is due to alternative binding to, and activation of, enhancer elements within target gene promoters. Nevertheless, the results are consistent with a global transcriptomic study in mouse embryonic fibroblasts (MEFs), which showed regulation of distinct gene sets in genetic models of Nrf2 deletion and induction [46].

CDDO and its various derivatives have been shown to affect several different intracellular signalling pathways, including NF-κB [27,28], STAT [24], ERK/SMAD [47,48] and PPARγ [49]. A particular focus of this study was to compare CDDO-me induced protein expression changes in Nrf2 competent and deficient mice. This allows us to define any changes observed as Nrf2 dependent or independent effects. Somewhat surprisingly, very few of the proteins induced by CDDO-me in wild type mice were similarly changed in the Nrf2^(−/−)^ animals, indicating that at the relatively low acute dose of CDDO-me used here, nearly all of the protein changes were mediated via the Keap1:Nrf2 signalling pathway.

With respect to defence against chemical toxins, a total of 65 proteins were detected that are directly involved in drug metabolism (including CYPs, UDP-GTs, epoxide hydrolase and glutathione transferases) and of these 21 were regulated by Nrf2 at the basal level and 9 following treatment with CDDO-me: only 5 drug metabolizing proteins were regulated both basally and after induction. These data indicate that the protein expression profile of Nrf2-regulated gene products is finely tuned to deal with exposure to small chemical xenobiotics that cause oxidative stress or the formation of protein reactive electrophilies. As was shown in our previous proteomic investigation of Nrf2^(−/−)^ mice, lipid metabolism featured strongly in the differentially regulated proteins, confirming a key role for Nrf2 in the modulation of fatty acid synthesis. The mechanism underlying this effect is not clear, since the expression of fatty acid synthetic enzymes is inversely related to Nrf2 activity, but may involve an interaction with other regulators of lipid metabolism such as PPARγ or sterol regulatory element-binding protein 1c (SREBP1c). An effect of Nrf2 on expression of genes involved in lipid metabolism has been noted at the mRNA level in several other studies. Mice fed a high fat diet expressed genes encoding enzymes key for fatty acid synthesis at a significantly higher level in Nrf2^(−/−)^ mice when compared to wild type animals [37]. A similar effect was observed at the mRNA level in an elegant study by Yates et al. [33] which compared hepatic transcription profiles in mice following exposure to another derivative of CDDO, CDDO-im, and in mice deficient in Keap1, which thus had constitutively activated Nrf2. Both chemical and genetic methods of Nrf2 induction resulted in the down-regulation of pivotal enzymes in the fatty acid pathway, such as ACL. Interestingly, in the current study, whilst wild type mice clearly under-expressed these proteins compared with Nrf2^(−/−)^ mice, treatment of wild type animals with CDDO-me did not result in a further decrease in expression. Thus the effects observed by Yates et al. at the mRNA level may not translate into altered expression at the protein level. A similar effect of CDDO-im was reported by Shin et al. [38] who also noted down regulation of fatty acid related genes using RT-PCR; however, this study involved prolonged treatment with CDDO-im over several weeks and an effect on lipid metabolism was not seen in mice after acute treatment with the inducer. Nevertheless, it is clear that Nrf2 has an important role for maintenance of lipid homeostasis in the liver; however, it appears from this study that the influence of Nrf2 at the basal level is more important with respect to lipid metabolism than the effect of induction.

Of the proteins that were up-regulated in CDDO-me treated wild type mice, CYP2A5 showed the greatest increase in protein expression. Nrf2-regulation of CYP2A5 has previously been documented [39,50], while studies employing human hepatocytes have also identified CYP2A6, the human analogue, as Nrf2 regulated [51]. Interestingly, CYP2A5/6 is important for the metabolism of compounds including coumarin, nicotine and caffeine, with products of coumarin and caffeine metabolism being employed as markers of enzyme activity [52,53]. Recently, CYP2A5 has also been shown to be involved in bilirubin clearance, and Nrf2-mediated regulation of CYP2A5 has been identified as having a role in the cytoprotective response to bilirubin-associated hepatotoxicity [54]. This may point to an evolutionary role for Nrf2 in defence against bilirubin toxicity through the co-ordinated phase I and II regulation metabolism.

ENTPD5 was another of the five proteins that was expressed at a significantly higher level in wild type mice treated with CDDO-me, as well as at a constitutively lower level in Nrf2^(−/−)^ animals. To our knowledge, Nrf2-mediated regulation of ENTPD5 at the protein level has not previously been documented, although an association has been identified at the mRNA level. *Entpd5* was shown to be upregulated in microarray analysis of a Keap1-deficient hepatocyte-specific mouse model [55] and its regulation at both the constitutive and inducible levels is consistent with a microarray/ChIP-Seq study that identified Nrf2 target genes in genetically modified MEFs [46]. ENTPD5 is becoming recognised as a pivotal protein in the respiratory switch to aerobic glycolysis, the Warburg effect, in many tumours [56]. ENTPD5 is a uridine diphosphatase that hydrolyzes uridine diphosphate (UDP) to uridine monophosphate (UMP). It is important in the glycosylation and folding of proteins, as well as in ATP regulation. It has been shown to play a role in regulation of the PI3K-PTEN-AKT signalling loop [57]. Interestingly, the PI3K/AKT signalling pathway has been implicated in Nrf2 signalling, notably in the triterpenoid-mediated activation of Nrf2 [18]. Furthermore, ENTPD5 deficient transgenic mice show an unusual phenotypic pathology comprising swelling of the hepatocytes within the centrilobular region, progressing to hepatocellular neoplasia with time [58].

In conclusion, this study provides the first comprehensive proteomic analysis of Nrf2-regulated liver protein expression at the constitutive and CDDO-me inducible level. Whilst both basal and inducible changes were observed, there was little overlap between the two lists of proteins quantified. Nevertheless, the most prominent groups of proteins under both conditions were those involved in the metabolism of xenobiotics, and thus, the study provides a clear rationale for the role of Nrf2 in protection against chemical toxins following both acute and chronic exposure. The definition of the Nrf2 inducible proteome in all organs, from a qualitative and quantitative perspective, would provide a useful platform for the development of Nrf2 inducers as therapeutic agents for the treatment of diseases which have aetiologies based on either chemical stress or chemical exposure. Furthermore, such proteomic analysis should provide a basis for the discovery of biomarkers which can be used to facilitate the translation of basic biochemical science into clinical practice.

The following are the supplementary data related to this article.

Supplementary Table 1 iTRAQ-based proteomic comparison of liver proteins in Nrf2^(−/−)^ and wild type mice. Proteins whose expression was different (*P* < 0.05) between Nrf2^(−/−)^ and wild type mice. Mean expression values relative to a common pool are given for *n* = 4–6 animals. Proteins are ordered according to the ratio between wild type and Nrf2^(−/−)^ mice (Nrf2^(+/+)^/Nrf2^(−/−)^; highest to lowest) such that proteins whose expression is most markedly reduced in Nrf2^(−/−)^ animals appear at the top of the list.
^a^Average number of peptides used for quantification across the four individual iTRAQ runs.

Supplementary Table 2 iTRAQ-based proteomic comparison of liver proteins in vehicle treated and CDDO-me treated wild type mice. Proteins listed are those whose expression was different (*P* < 0.05) between wild type (Nrf2^(+/+)^) and wild type CDDO-me (Nrf2^(+/+)^CDDO) treated mice. Mean expression values relative to a common pool are given for *n* = 4–6 animals. Proteins are ordered according to the ratio between CDDO-me treated wild type mice and vehicle control treated wild type mice (Nrf2^(+/+)^CDDO/Nrf2^(+/+)^; highest to lowest) such that proteins whose expression is most markedly induced by CDDO-me appear at the top of the list.
^a^Average number of peptides used for quantification across the four individual iTRAQ runs.

Supplementary Table 3 iTRAQ-based proteomic comparison of liver proteins in vehicle control and CDDO-me treated Nrf2^(−/−)^ mice. Proteins listed are those whose expression was different (*P* < 0.05) between Nrf2^(−/−)^ and Nrf2^(−/−)^-CDDO-me treated mice. Mean expression values relative to a common pool are given for *n* = 4–6 animals. Proteins are ordered according to the ratio between CDDO-me treated Nrf2^(−/−)^ mice and vehicle treated Nrf2^(−/−)^ mice (Nrf2^(−/−)^CDDO/Nrf2^(−/−)^; highest to lowest) such that proteins whose expression is most markedly induced by CDDO-me appear at the top of the list.
^a^Average number of peptides used for quantification across the four individual iTRAQ runs.

Supplementary Table 4 Enhancer element binding site analysis of the proteins regulated by Nrf2 at both the constitutive and inducible levels. Full length gene and promoter (3000 bp) DNA sequences were interrogated for consensus Nrf2 binding sites using the MatInspector search in the Genomatix software suite, focussing on the MAF and AP1 related factor subgroup (V$AP1R). Full length gene sequences were retrieved from Entrez gene, while promoter sequences were extracted using the Gene2Promoter tool of the Genomatix software. Matrix similarity was optimised and core similarity was set to 0.75. Results presented in the table are limited to sequences associated with three transcription factor binding sites: NF-E2 p45, antioxidant response elements and binding sites for heterodimers with small Maf-proteins.

Supplementary data to this article can be found online at http://dx.doi.org/10.1016/j.jprot.2014.05.007.

## Transparency Document

Transparency Document.

## Transparency Document

Transparency Document associated with this article can be found, in the online version.

## Figures and Tables

**Table 1:** Constitutively regulated and CDDO-me inducible proteins. iTRAQ-based proteomic comparison of liver proteins in vehicle treated and CDDO-me treated Nrf2^(−/−)^ and wild type mice. Proteins whose expression was down-regulated or up-regulated by at least 30% (*P* < 0.05) in vehicle treated Nrf2^(−/−)^ relative to wild type mice, in wild type mice following CDDO-me administration or in Nrf2^(−/−)^ mice following CDDO-me administration are listed. Mean expression values relative to a common pool are given for *n* = 4–6 animals. In the first instance, proteins are ordered according to the ratio between wild type and Nrf2^(−/−)^ mice (Nrf2^(+/+)^/Nrf2^(−/−)^; highest to lowest) such that proteins whose expression is most markedly constitutively reduced in Nrf2^(−/−)^ animals appear at the top of the list. Remaining proteins are then ordered according to the ratio between CDDO-me treated wild type mice and vehicle treated wild type mice (Nrf2^(+/+)^CDDO/Nrf2^(+/+)^; highest to lowest) such that proteins whose expression is most markedly induced by CDDO-me in wild type mice appear highest in the list. Finally, proteins are ordered according to the ratio between CDDO-me treated Nrf2^(−/−)^ mice and vehicle treated Nrf2^(−/−)^ mice (Nrf2^(−/−)^ CDDO/Nrf2^(−/−)^; highest to lowest) such that proteins whose expression is most markedly induced by CDDO-me in Nrf2^(−/−)^ mice appear highest in the list. Complete lists of all significantly altered proteins in wild type relative to Nrf2^(−/−)^ mice and all proteins significantly altered by CDDO-me in wild type and Nrf2^(−/−)^ mice are given in Supplementary Tables 1, 2 and 3 respectively.

UniProt accession	Name	Peptides^a^	Nrf2^(+/+)^ Nrf2^(−/−)^	*P* value	Nrf2^(+/+)^CDDO Nrf2^(+/+)^	*P* value	Nrf2^(−/−)^CDDO Nrf2^(−/−)^	*P* value
P17717	UDP-glucuronosyltransferase 2B17	38	4.28	< 0.001				
P10649	Glutathione S-transferase Mu 1	69	4.11	< 0.001	1.43	0.022		
P19639	Glutathione S-transferase Mu 3	49	4.04	< 0.001	1.58	< 0.001		
P02762	Major urinary protein 6	35	3.62	< 0.001				
O70475	UDP-glucose 6-dehydrogenase	24	2.64	< 0.001	1.57	< 0.001		
Q8VCC2	Liver carboxylesterase 1	13	2.64	0.030				
P97493	Thioredoxin, mitochondrial	4	2.52	0.026				
P30115	Glutathione S-transferase A3	30	2.42	0.007				
Q9WUZ9	Ectonucleoside triphosphate diphosphohydrolase 5	8	2.22	0.014	2.04	< 0.001		
P24549	Retinal dehydrogenase 1	68	2.17	< 0.001				
O08709	Peroxiredoxin-6	34	2.16	< 0.001				
P20852	Cytochrome P450 2A5	11	2.12	0.046	8.12	< 0.001		
P19157	Glutathione S-transferase P 1	124	2.12	0.002				
P15626	Glutathione S-transferase Mu 2	37	2.09	< 0.001				
Q60991	25-hydroxycholesterol 7-alpha-hydroxylase	11	2.09	0.012				
P22907	Porphobilinogen deaminase	7	2.04	< 0.001				
Q9D379	Epoxide hydrolase 1	14	2.00	0.001	1.48	0.002		
P06801	NADP-dependent malic enzyme	35	1.91	< 0.001				
Q6XVG2	Cytochrome P450 2C54	16	1.88	< 0.001	0.67	0.009		
Q91X77	Cytochrome P450 2C50	21	1.79	0.002				
Q9CXN7	Phenazine biosynthesis-like domain-containing protein 2	15	1.70	0.001				
Q8R0Y6	Cytosolic 10-formyltetrahydrofolate dehydrogenase	113	1.70	0.001				
Q9D1L0	Coiled-coil-helix-coiled-coil-helix domain-containing protein 2, mitochondrial	4	1.69	0.016				
Q63836	Selenium-binding protein 2	131	1.67	0.040				
Q9DBG1	Sterol 26-hydroxylase, mitochondrial	29	1.66	0.026				
Q9DCY0	Glycine N-acyltransferase-like protein Keg1	12	1.65	0.001			0.56	0.021
Q91VA0	Acyl-coenzyme A synthetase ACSM1, mitochondrial	42	1.64	< 0.001				
O88487	Cytoplasmic dynein 1 intermediate chain 2	4	1.63	0.003				
Q9QZX7	Serine racemase	1	1.62	0.002				
Q91VS7	Microsomal glutathione S-transferase 1	32	1.62	0.046				
Q8VC30	Bifunctional ATP-dependent dihydroxyacetone kinase/FAD-AMP lyase (cyclizing)	77	1.60	0.039				
P24472	Glutathione S-transferase A4	8	1.58	0.003				
Q64442	Sorbitol dehydrogenase	36	1.58	< 0.001				
Q64458	Cytochrome P450 2C29	26	1.56	0.029				
P52760	Ribonuclease UK114	35	1.55	< 0.001				
O70570	Polymeric immunoglobulin receptor	3	1.55	< 0.001				
Q9EQK5	Major vault protein	14	1.55	< 0.001				
Q922Q8	Leucine-rich repeat-containing protein 59	10	1.52	0.008				
Q8CG76	Aflatoxin B1 aldehyde reductase member 2	10	1.51	< 0.001				
O55022	Membrane-associated progesterone receptor component 1	9	1.50	0.019				
Q80W22	Threonine synthase-like 2	8	1.49	0.002				
P61922	4-aminobutyrate aminotransferase, mitochondrial	28	1.49	0.036				
Q9DCM0	Protein ETHE1, mitochondrial	8	1.48	0.003				
Q91V76	Ester hydrolase C11orf54 homolog	13	1.48	0.002				
P15105	Glutamine synthetase	34	1.46	0.031				
Q14CH1	Molybdenum cofactor sulfurase	2	1.46	0.025				
O88844	Isocitrate dehydrogenase [NADP] cytoplasmic	55	1.46	0.023				
Q9R0P3	S-formylglutathione hydrolase	19	1.45	0.002				
P63101	14-3-3 protein zeta/delta	21	1.43	0.015				
P50431	Serine hydroxymethyltransferase, cytosolic	19	1.43	0.016				
P70398	Probable ubiquitin carboxyl-terminal hydrolase FAF-X	7	1.42	0.041	0.64	0.011		
O08966	Solute carrier family 22 member 1	2	1.42	0.034				
Q91X52	l-xylulose reductase	7	1.41	0.035				
Q9JII6	Alcohol dehydrogenase [NADP +]	20	1.39	0.012				
Q8K1N1	Calcium-independent phospholipase A2-gamma	3	1.39	0.023				
P47738	Aldehyde dehydrogenase, mitochondrial	119	1.38	0.036				
Q91YP3	Putative deoxyribose-phosphate aldolase	4	1.38	0.029				
Q9DBG5	Perilipin-3	5	1.37	0.021				
Q64514	Tripeptidyl-peptidase 2	10	1.37	0.012				
Q64737	Trifunctional purine biosynthetic protein adenosine-3	8	1.37	0.042				
Q8VCA8	Secernin-2	13	1.36	0.022				
P28474	Alcohol dehydrogenase class-3	24	1.35	0.018				
Q3UJU9	Regulator of microtubule dynamics protein 3	7	1.35	< 0.001				
Q8K157	Aldose 1-epimerase	8	1.33	0.014				
Q9Z2W0	Aspartyl aminopeptidase	7	1.32	0.013				
Q99KQ4	Nicotinamide phosphoribosyltransferase	6	1.31	0.049				
Q9WU79	Proline dehydrogenase 1, mitochondrial	22	1.30	0.046				
Q9JMH6	Thioredoxin reductase 1, cytoplasmic	7	1.30	0.037				
Q8C854	Myelin expression factor 2	1	0.70	0.002				
Q811U4	Mitofusin-1	2	0.70	0.034				
Q9D2G2	Dihydrolipoyllysine-residue succinyltransferase component of 2-oxoglutarate dehydrogenase complex, mitochondrial	14	0.70	0.049				
Q64FW2	All-trans-retinol 13,14-reductase	8	0.69	0.041			0.55	0.004
P48678	Prelamin-A/C	18	0.68	0.002				
Q4VBD2	Transmembrane anterior posterior transformation protein 1	1	0.68	0.012			0.61	0.002
P25688	Uricase	36	0.67	0.027				
Q8VEH5	EPM2A-interacting protein 1	2	0.67	0.019				
P08032	Spectrin alpha chain, erythrocyte	7	0.66	0.005				
P21981	Protein-glutamine gamma-glutamyltransferase 2	16	0.66	0.049				
Q9WU19	Hydroxyacid oxidase 1	9	0.65	< 0.001				
Q6ZWY9	Histone H2B type 1-C/E/G	30	0.65	0.020				
O08917	Flotillin-1	2	0.63	0.002			0.69	0.018
P32020	Non-specific lipid-transfer protein	72	0.62	0.003				
Q99P30	Peroxisomal coenzyme A diphosphatase NUDT7	28	0.62	0.008				
Q9CQC9	GTP-binding protein SAR1b	15	0.60	0.010			0.63	0.017
P11714	Cytochrome P450 2D9	42	0.58	0.008				
Q05816	Fatty acid-binding protein, epidermal	8	0.40	0.003				
O35728	Cytochrome P450 4A14	7	0.39	0.018			0.42	0.023
Q8JZK9	Hydroxymethylglutaryl-CoA synthase, cytoplasmic	10			1.76	0.016		
Q8C165	Probable carboxypeptidase PM20D1	4			1.64	0.049	1.73	0.047
P48758	Carbonyl reductase [NADPH] 1	18			1.63	< 0.001		
Q9QYF1	Retinol dehydrogenase 11	2			1.52	0.042		
P58044	Isopentenyl-diphosphate Delta-isomerase 1	3			1.48	0.037	1.63	0.015
P50285	Dimethylaniline monooxygenase [N-oxide-forming] 1	18			1.45	0.001		
Q9R1J0	Sterol-4-alpha-carboxylate 3-dehydrogenase, decarboxylating	8			1.42	0.034		
Q07076	Annexin A7	4			1.42	0.003		
P38060	Hydroxymethylglutaryl-CoA lyase, mitochondrial	21			1.34	0.017		
Q9DD20	Methyltransferase-like protein 7B	15			1.33	0.015		
Q923D2	Flavin reductase (NADPH)	9			1.33	0.004		
P29341	Polyadenylate-binding protein 1	18			1.32	0.014		
Q91Y97	Fructose-bisphosphate aldolase B	111			0.69	0.037		
P70255	Nuclear factor 1 C-type	1			0.61	0.012		
P09103	Protein disulfide-isomerase	83					1.53	0.028
Q8VCM7	Fibrinogen gamma chain	11					1.52	< 0.001
P62082	40S ribosomal protein S7	17					1.49	0.013
P19324	Serpin H1	3					1.47	0.044
Q9DBG7	Signal recognition particle receptor subunit alpha	4					1.47	0.019
P24369	Peptidyl-prolyl cis-trans isomerase B	8					1.46	0.017
Q8QZZ7	TP53RK-binding protein	1					1.46	0.042
P27773	Protein disulfide-isomerase A3	49					1.44	0.016
O08600	Endonuclease G, mitochondrial	3					1.40	0.004
Q9JHK4	Geranylgeranyl transferase type-2 subunit alpha	2					1.40	0.010
P18760	Cofilin-1	14					1.39	0.013
Q922E4	Ethanolamine-phosphate cytidylyltransferase	9					1.37	0.012
Q8BW75	Amine oxidase [flavin-containing] B	18					1.37	0.013
P49722	Proteasome subunit alpha type-2	11					1.37	0.024
P99027	60S acidic ribosomal protein P2	19					1.36	0.020
Q9CQF9	Prenylcysteine oxidase	5					1.36	0.036
P14211	Calreticulin	24					1.36	0.038
O08795	Glucosidase 2 subunit beta	6					1.34	0.001
Q921M3	Splicing factor 3B subunit 3	4					1.34	0.009
O70503	Estradiol 17-beta-dehydrogenase 12	6					1.33	0.032
Q9DCM2	Glutathione S-transferase kappa 1	9					1.33	0.040
P62702	40S ribosomal protein S4, X isoform	13					1.33	0.043
P47962	60S ribosomal protein L5	13					1.32	0.024
Q60866	Phosphotriesterase-related protein	7					1.31	0.006
Q9CXI5	Mesencephalic astrocyte-derived neurotrophic factor	4					1.30	0.033
P62827	GTP-binding nuclear protein Ran	6					1.30	0.042
Q3ULD5	Methylcrotonoyl-CoA carboxylase beta chain, mitochondrial	14					0.58	< 0.001
O09158	Cytochrome P450 3A25	4					0.54	0.032
Q64459	Cytochrome P450 3A11	27					0.46	< 0.001
Q99LY9	NADH dehydrogenase [ubiquinone] iron-sulfur protein 5	2					0.46	0.031
O88833	Cytochrome P450 4A10	9					0.29	0.012
O35386	Phytanoyl-CoA dioxygenase, peroxisomal	5					1.70	0.008
Q91WL5	Cytochrome P450 4A12A	17					0.60	0.024
P61924	Coatomer subunit zeta-1	1					0.67	0.040
Q62189	U1 small nuclear ribonucleoprotein A	1					0.67	0.017
P55050	Fatty acid-binding protein, intestinal	2					0.69	0.028
Q99L13	3-hydroxyisobutyrate dehydrogenase, mitochondrial	14					0.69	0.041
Q9Z0M5	Lysosomal acid lipase/cholesteryl ester hydrolase	5					0.70	0.031

a Average number of peptides used for quantification across the four individual iTRAQ runs.

**Table 2:** Pathway analysis of Nrf2-regulated gene products at the basal level. GeneGo Metacore was used to identify pathways enriched in the wild type animals compared with the Nrf2^(−/−)^ mice. All significant (*P* < 0.05) pathways are listed along with the number of objects within the protein set associated with that pathway. The total number of objects in the entire pathway is shown in parentheses.

	Pathway	*P* value	Objects
1	Pyruvate metabolism/rodent version	0.0000040	7 (66)
2	NRF2 regulation of oxidative stress response	0.000016	6 (54)
3	Naphthalene metabolism	0.000032	6 (61)
4	Glutathione metabolism/rodent version	0.000075	6 (71)
5	Glutathione metabolism	0.00048	5 (65)
6	Glutathione metabolism/human version	0.00051	5 (66)
7	Tryptophan metabolism/rodent version	0.00055	6 (102)
8	CAR-mediated direct regulation of xenobiotic metabolizing enzymes/rodent version	0.00074	4 (41)
9	CAR-mediated direct regulation of xenobiotic metabolizing enzymes/human version	0.00074	4 (41)
10	Pyruvate metabolism	0.0015	4 (49)
11	Lysine metabolism/rodent version	0.0018	5 (87)
12	Transcription_Transcription regulation of aminoacid metabolism	0.0019	3 (25)
13	Folic acid metabolism	0.0019	4 (53)
14	Triacylglycerol metabolism p.1	0.0031	4 (60)
15	Tryptophan metabolism	0.0035	5 (101)
16	Ascorbate metabolism/rodent version	0.0036	3 (31)
17	Butanoate metabolism	0.0037	4 (63)
18	Development_EPO-induced Jak-STAT pathway	0.0051	3 (35)
19	Retinol metabolism/rodent version	0.0053	4 (70)
20	Transcription_Role of AP-1 in regulation of cellular metabolism	0.0065	3 (38)
21	Retinol metabolism	0.0065	4 (74)
22	Propionate metabolism p.1	0.0070	3 (39)
23	Histidine-glutamate-glutamine and proline metabolism/rodent version	0.0072	5 (120)
24	Leucine, isoleucine and valine metabolism/rodent version	0.0085	4 (80)
25	Benzo[a]pyrene metabolism	0.0086	3 (42)
26	Immune response_IL-7 signaling in B lymphocytes	0.0092	3 (43)
27	Immune response_IL-5 signalling	0.0098	3 (44)
28	Lysine metabolism	0.011	4 (85)
29	Mechanisms of CFTR activation by S-nitrosoglutathione (normal and CF)	0.011	3 (46)
30	Androstenedione and testosterone biosynthesis and metabolism p.1	0.016	3 (53)
31	Immune response_Fc epsilon RI pathway	0.018	3 (55)
32	Androstenedione and testosterone biosynthesis and metabolism p.1/rodent version	0.020	3 (57)
33	Immune response_CCR5 signaling in macrophages and T lymphocytes	0.021	3 (58)
34	Propionate metabolism p.2	0.029	3 (66)
35	Polyamine metabolism	0.031	3 (68)
36	Acetaminophen metabolism	0.034	2 (29)
37	Histamine metabolism	0.034	2 (29)
38	Immune response_Signaling pathway mediated by IL-6 and IL-1	0.036	2 (30)
39	Cholesterol and sphingolipids transport/distribution to the intracellular membrane compartments (normal and CF)	0.039	2 (31)
40	Beta-alanine metabolism/rodent version	0.041	2 (32)
41	Signal transduction_ERK1/2 signaling pathway	0.041	2 (32)
42	(L)-Arginine metabolism	0.041	3 (76)
43	Leucine, isoleucine and valine metabolism.p.2	0.044	3 (78)
44	Development_CNTF receptor signalling	0.046	2 (34)
45	Fatty acid omega oxidation	0.046	2 (34)
46	Immune response_Role of the Membrane attack complex in cell survival	0.046	2 (34)
47	Immune response_Oncostatin M signaling via MAPK in mouse cells	0.048	2 (35)
48	Estrone metabolism	0.048	2 (35)

**Table 3:** Pathway analysis of Nrf2-regulated gene products induced by CDDO-me. GeneGo Metacore was used to identify pathways enriched in the wild type animals treated with CDDO-me (3 mg/kg) for 24 h compared with the vehicle treated wild type mice. All significant (*P* < 0.05) pathways are listed along with the number of objects within the protein set associated with that pathway. The total number of objects in the entire pathway is shown in parentheses.

	Pathway	*P* value	Objects
1	Glycolysis and gluconeogenesis (short map)	0.0015	3 (66)
2	Cholesterol biosynthesis	0.0034	3 (88)
3	Glycogen metabolism	0.0076	2 (38)
4	SCAP/SREBP transcriptional control of cholesterol and FA biosynthesis	0.0084	2 (40)
5	Galactose metabolism	0.018	2 (59)
6	Fructose metabolism	0.027	2 (74)
7	Peroxisomal branched chain fatty acid oxidation	0.033	2 (83)
8	Fructose metabolism/rodent version	0.034	2 (84)

**Table 4:** Proteins regulated by Nrf2 at both basal and CDDO-me-inducible levels. iTRAQ-based proteomic comparison of liver proteins in vehicle control treated wild type and Nrf2^(−/−)^ mice and CDDO-me treated wild type mice*.* Proteins whose expression was up- or down-regulated by at least 30% at both the basal and CDDO-me-inducible level are listed. Mean expression values relative to a common pool are given for *n* = 4–6 mice. Proteins are ordered according to the ratio between CDDO-me treated wild type and Nrf2^(−/−)^ mice (Nrf2^(+/+)^ CDDO/Nrf2^(−/−)^; highest to lowest), such that proteins showing the widest range of Nrf2 regulation appear at the top of the list.

UniProt accession	Name	Nrf2^(+/+)^CDDO Nrf2^(−/−)^ ctrl	Protein function^a^
P20852	Cytochrome P450 2A5	**17.24**	Cytochrome P450 exhibiting high coumarin 7-hydroxylase activity.
P19639	Glutathione S-transferase Mu 3	**6.39**	Mediates the conjugation of GSH to a wide number of exogenous and endogenous electrophiles.
P10649	Glutathione S-transferase Mu 1	**5.86**	Mediates the conjugation of GSH to a wide number of exogenous and endogenous electrophiles.
Q9WUZ9	Ectonucleoside triphosphate diphosphohydrolase 5	**4.55**	Uridine diphosphatase that promotes protein N-glycosylation and ATP regulation. With CMPK1 and AK1, constitutes an ATP hydrolysis cycle converting ATP to AMP resulting in a compensatory increase in aerobic glycolysis. Plays a key role in the AKT1-PTEN signalling pathway by promoting glycolysis in proliferating cells in response to PI3K signalling.
O70475	UDP-glucose 6-dehydrogenase	**4.14**	Involved in the biosynthesis of UDPGA, glycosaminoglycans, hyaluronan, chondroitin sulfate, and heparan sulphate.
Q9D379	Epoxide hydrolase 1	**2.96**	Enzyme that catalyzes the hydrolysis of arene and aliphatic epoxides to less reactive and more water soluble dihydrodiols by the trans addition of water.

a Protein function based on the UniProt database annotation (http://www.uniprot.org/).
