# Supplementary material for: Identification and quantification of the basal and inducible Nrf2-dependent proteomes in mouse liver: Biochemical, pharmacological and toxicological implications
Source: J Proteomics. 2014 Aug 28;108(100):171–87. doi: 10.1016/j.jprot.2014.05.007 (PMC4115266; doi:10.1016/j.jprot.2014.05.007)
Supplement: Supplementary Table 1 — iTRAQ-based proteomic comparison of liver proteins in Nrf2(−/−) and wild type mice. Proteins whose expression was different (P < 0.05) between Nrf2(−/−) and wild type mice. Mean expression values relative to a common pool are given for n = 4–6 animals. Proteins are ordered according to the ratio between wild type and Nrf2(−/−) mice (Nrf2(+/+)/Nrf2(−/−); highest to lowest) such that proteins whose expression is most markedly reduced in Nrf2(−/−) animals appear at the top of the list. aAverage number of peptides used for quantification across the four individual iTRAQ runs. [file mmc1.docx]

**Supplementary Table 1:**  *iTRAQ-based proteomic comparison of liver proteins in Nrf2^(-/-)^ and wild type mice.* Proteins whose expression was different (P < 0.05) between Nrf2^(-/-)^ and wild type mice. Mean expression values relative to a common pool are given for n=4-6 animals. Proteins are ordered according to the ratio between wild type and Nrf2^(-/-)^ mice (Nrf2^(+/+)^/Nrf2^(-/-)^; highest to lowest) such that proteins whose expression is most markedly reduced in Nrf2^(-/-)^ animals appear at the top of the list.

*^a^*Average number of peptides used for quantification across the four individual iTRAQ runs.

| **Uniprot** |  |  |  | **Nrf2^(+/+)^** | | |  | **Nrf2^(-/-)^** | | |  | **Nrf2^(+/+)^ Nrf2^(-/-)^** |  |
| --- | --- | --- | --- | --- | --- | --- | --- | --- | --- | --- | --- | --- | --- |
| **Accession** | **Name** | **Peptides***^a^* |  | **mean** | **n** | **SD** |  | **mean** | **n** | **SD** |  | **ratio** | **P-value** |
| P17717 | UDP-glucuronosyltransferase 2B17 | 38 |  | 1.58 | 6 | 0.54 |  | 0.37 | 6 | 0.19 |  | 4.28 | <0.001 |
| P10649 | Glutathione S-transferase Mu 1 | 69 |  | 1.03 | 6 | 0.29 |  | 0.25 | 6 | 0.10 |  | 4.11 | <0.001 |
| P19639 | Glutathione S-transferase Mu 3 | 49 |  | 1.33 | 6 | 0.19 |  | 0.33 | 6 | 0.22 |  | 4.04 | <0.001 |
| P02762 | Major urinary protein 6 | 35 |  | 1.55 | 6 | 0.74 |  | 0.43 | 6 | 0.23 |  | 3.62 | <0.001 |
| O70475 | UDP-glucose 6-dehydrogenase | 24 |  | 1.19 | 6 | 0.36 |  | 0.45 | 6 | 0.22 |  | 2.64 | <0.001 |
| Q8VCC2 | Liver carboxylesterase 1 | 13 |  | 2.08 | 6 | 1.04 |  | 0.79 | 6 | 0.73 |  | 2.64 | 0.030 |
| P97493 | Thioredoxin, mitochondrial | 4 |  | 2.36 | 6 | 1.63 |  | 0.94 | 6 | 0.08 |  | 2.52 | 0.026 |
| P30115 | Glutathione S-transferase A3 | 30 |  | 1.21 | 6 | 0.39 |  | 0.50 | 6 | 0.23 |  | 2.42 | 0.007 |
| Q9WUZ9 | Ectonucleoside triphosphate diphosphohydrolase 5 | 8 |  | 1.05 | 6 | 0.19 |  | 0.47 | 6 | 0.16 |  | 2.22 | 0.014 |
| P24549 | Retinal dehydrogenase 1 | 68 |  | 1.40 | 6 | 0.40 |  | 0.65 | 6 | 0.19 |  | 2.17 | <0.001 |
| O08709 | Peroxiredoxin-6 | 34 |  | 1.59 | 6 | 0.58 |  | 0.74 | 6 | 0.16 |  | 2.16 | <0.001 |
| P19157 | Glutathione S-transferase P 1 | 124 |  | 1.33 | 6 | 0.41 |  | 0.63 | 6 | 0.32 |  | 2.12 | 0.002 |
| P20852 | Cytochrome P450 2A5 | 11 |  | 0.40 | 6 | 0.10 |  | 0.19 | 6 | 0.11 |  | 2.12 | 0.046 |
| P15626 | Glutathione S-transferase Mu 2 | 37 |  | 1.39 | 6 | 0.36 |  | 0.67 | 6 | 0.18 |  | 2.09 | <0.001 |
| Q60991 | 25-hydroxycholesterol 7-alpha-hydroxylase | 11 |  | 1.11 | 6 | 0.44 |  | 0.53 | 6 | 0.20 |  | 2.09 | 0.012 |
| P22907 | Porphobilinogen deaminase | 7 |  | 1.32 | 6 | 0.11 |  | 0.65 | 5 | 0.07 |  | 2.04 | <0.001 |
| Q9D379 | Epoxide hydrolase 1 | 14 |  | 1.25 | 6 | 0.37 |  | 0.63 | 6 | 0.12 |  | 2.00 | 0.001 |
| P06801 | NADP-dependent malic enzyme | 35 |  | 1.30 | 6 | 0.34 |  | 0.68 | 6 | 0.14 |  | 1.91 | <0.001 |
| Q6XVG2 | Cytochrome P450 2C54 | 16 |  | 1.41 | 6 | 0.29 |  | 0.75 | 6 | 0.36 |  | 1.88 | <0.001 |
| Q91X77 | Cytochrome P450 2C50 | 21 |  | 1.30 | 6 | 0.21 |  | 0.73 | 5 | 0.20 |  | 1.79 | 0.002 |
| Q9CXN7 | Phenazine biosynthesis-like domain-containing protein 2 | 15 |  | 1.29 | 6 | 0.32 |  | 0.76 | 5 | 0.22 |  | 1.70 | 0.001 |
| Q8R0Y6 | Cytosolic 10-formyltetrahydrofolate dehydrogenase | 113 |  | 1.32 | 6 | 0.38 |  | 0.77 | 6 | 0.10 |  | 1.70 | 0.001 |
| Q9D1L0 | Coiled-coil-helix-coiled-coil-helix domain-containing protein 2, mitochondrial | 4 |  | 1.22 | 6 | 0.32 |  | 0.72 | 5 | 0.24 |  | 1.69 | 0.016 |
| Q63836 | Selenium-binding protein 2 | 131 |  | 1.30 | 6 | 0.56 |  | 0.78 | 6 | 0.31 |  | 1.67 | 0.040 |
| Q9DBG1 | Sterol 26-hydroxylase, mitochondrial | 29 |  | 1.21 | 5 | 0.16 |  | 0.73 | 4 | 0.43 |  | 1.66 | 0.026 |
| Q9DCY0 | Glycine N-acyltransferase-like protein Keg1 | 12 |  | 1.76 | 6 | 0.27 |  | 1.07 | 6 | 0.26 |  | 1.65 | 0.001 |
| Q91VA0 | Acyl-coenzyme A synthetase ACSM1, mitochondrial | 42 |  | 1.23 | 6 | 0.06 |  | 0.75 | 6 | 0.14 |  | 1.64 | <0.001 |
| O88487 | Cytoplasmic dynein 1 intermediate chain 2 | 4 |  | 1.00 | 5 | 0.16 |  | 0.61 | 4 | 0.24 |  | 1.63 | 0.003 |
| Q9QZX7 | Serine racemase | 1 |  | 1.20 | 6 | 0.15 |  | 0.74 | 5 | 0.36 |  | 1.62 | 0.002 |
| Q91VS7 | Microsomal glutathione S-transferase 1 | 32 |  | 1.41 | 6 | 0.75 |  | 0.87 | 6 | 0.23 |  | 1.62 | 0.046 |
| Q8VC30 | Bifunctional ATP-dependent dihydroxyacetone kinase/FAD-AMP lyase (cyclizing) | 77 |  | 1.55 | 6 | 0.67 |  | 0.97 | 6 | 0.34 |  | 1.60 | 0.039 |
| P24472 | Glutathione S-transferase A4 | 8 |  | 1.28 | 6 | 0.24 |  | 0.81 | 6 | 0.18 |  | 1.58 | 0.003 |
| Q64442 | Sorbitol dehydrogenase | 36 |  | 1.33 | 6 | 0.22 |  | 0.84 | 6 | 0.17 |  | 1.58 | <0.001 |
| Q64458 | Cytochrome P450 2C29 | 26 |  | 1.41 | 6 | 0.35 |  | 0.91 | 6 | 0.42 |  | 1.56 | 0.029 |
| P52760 | Ribonuclease UK114 | 35 |  | 1.29 | 6 | 0.12 |  | 0.84 | 6 | 0.10 |  | 1.55 | <0.001 |
| O70570 | Polymeric immunoglobulin receptor | 3 |  | 1.19 | 5 | 0.22 |  | 0.77 | 4 | 0.05 |  | 1.55 | <0.001 |
| Q9EQK5 | Major vault protein | 14 |  | 1.14 | 6 | 0.08 |  | 0.74 | 6 | 0.16 |  | 1.55 | <0.001 |
| Q922Q8 | Leucine-rich repeat-containing protein 59 | 10 |  | 1.08 | 6 | 0.18 |  | 0.71 | 6 | 0.25 |  | 1.52 | 0.008 |
| Q8CG76 | Aflatoxin B1 aldehyde reductase member 2 | 10 |  | 1.21 | 6 | 0.12 |  | 0.80 | 6 | 0.12 |  | 1.51 | <0.001 |
| O55022 | Membrane-associated progesterone receptor component 1 | 9 |  | 1.07 | 6 | 0.13 |  | 0.71 | 6 | 0.14 |  | 1.50 | 0.019 |
| Q80W22 | Threonine synthase-like 2 | 8 |  | 1.29 | 6 | 0.21 |  | 0.87 | 6 | 0.22 |  | 1.49 | 0.002 |
| P61922 | 4-aminobutyrate aminotransferase, mitochondrial | 28 |  | 1.31 | 6 | 0.40 |  | 0.88 | 6 | 0.28 |  | 1.49 | 0.036 |
| Q9DCM0 | Protein ETHE1, mitochondrial | 8 |  | 1.65 | 6 | 0.41 |  | 1.11 | 6 | 0.23 |  | 1.48 | 0.003 |
| Q91V76 | Ester hydrolase C11orf54 homolog | 13 |  | 1.12 | 6 | 0.09 |  | 0.76 | 6 | 0.22 |  | 1.48 | 0.002 |
| P15105 | Glutamine synthetase | 34 |  | 1.15 | 6 | 0.18 |  | 0.79 | 6 | 0.32 |  | 1.46 | 0.031 |
| Q14CH1 | Molybdenum cofactor sulfurase | 2 |  | 1.05 | 6 | 0.17 |  | 0.72 | 5 | 0.34 |  | 1.46 | 0.025 |
| O88844 | Isocitrate dehydrogenase [NADP] cytoplasmic | 55 |  | 1.03 | 6 | 0.11 |  | 0.70 | 6 | 0.16 |  | 1.46 | 0.023 |
| Q9R0P3 | S-formylglutathione hydrolase | 19 |  | 1.19 | 6 | 0.14 |  | 0.82 | 6 | 0.15 |  | 1.45 | 0.002 |
| P63101 | 14-3-3 protein zeta/delta | 21 |  | 1.19 | 6 | 0.20 |  | 0.83 | 6 | 0.28 |  | 1.43 | 0.015 |
| P50431 | Serine hydroxymethyltransferase, cytosolic | 19 |  | 1.06 | 6 | 0.09 |  | 0.74 | 6 | 0.28 |  | 1.43 | 0.016 |
| P70398 | Probable ubiquitin carboxyl-terminal hydrolase FAF-X | 7 |  | 1.07 | 5 | 0.11 |  | 0.76 | 4 | 0.32 |  | 1.42 | 0.041 |
| O08966 | Solute carrier family 22 member 1 | 2 |  | 1.36 | 6 | 0.29 |  | 0.95 | 6 | 0.29 |  | 1.42 | 0.034 |
| Q91X52 | L-xylulose reductase | 7 |  | 1.24 | 6 | 0.28 |  | 0.88 | 5 | 0.35 |  | 1.41 | 0.035 |
| Q9JII6 | Alcohol dehydrogenase [NADP+] | 20 |  | 1.11 | 6 | 0.08 |  | 0.80 | 6 | 0.23 |  | 1.39 | 0.012 |
| Q8K1N1 | Calcium-independent phospholipase A2-gamma | 3 |  | 0.90 | 6 | 0.13 |  | 0.65 | 5 | 0.14 |  | 1.39 | 0.023 |
| P47738 | Aldehyde dehydrogenase, mitochondrial | 119 |  | 1.17 | 6 | 0.19 |  | 0.85 | 6 | 0.26 |  | 1.38 | 0.036 |
| Q91YP3 | Putative deoxyribose-phosphate aldolase | 4 |  | 1.22 | 6 | 0.15 |  | 0.88 | 5 | 0.30 |  | 1.38 | 0.029 |
| Q9DBG5 | Perilipin-3 | 5 |  | 1.22 | 5 | 0.17 |  | 0.90 | 4 | 0.23 |  | 1.37 | 0.021 |
| Q64514 | Tripeptidyl-peptidase 2 | 10 |  | 1.32 | 6 | 0.12 |  | 0.96 | 6 | 0.29 |  | 1.37 | 0.012 |
| Q64737 | Trifunctional purine biosynthetic protein adenosine-3 | 8 |  | 1.01 | 5 | 0.05 |  | 0.73 | 4 | 0.34 |  | 1.37 | 0.042 |
| Q8VCA8 | Secernin-2 | 13 |  | 1.44 | 6 | 0.38 |  | 1.05 | 6 | 0.32 |  | 1.36 | 0.022 |
| P28474 | Alcohol dehydrogenase class-3 | 24 |  | 1.09 | 6 | 0.14 |  | 0.81 | 6 | 0.17 |  | 1.35 | 0.018 |
| Q3UJU9 | Regulator of microtubule dynamics protein 3 | 7 |  | 1.22 | 6 | 0.06 |  | 0.90 | 6 | 0.12 |  | 1.35 | <0.001 |
| Q8K157 | Aldose 1-epimerase | 8 |  | 1.18 | 6 | 0.18 |  | 0.89 | 6 | 0.20 |  | 1.33 | 0.014 |
| Q9Z2W0 | Aspartyl aminopeptidase | 7 |  | 1.12 | 6 | 0.17 |  | 0.85 | 6 | 0.16 |  | 1.32 | 0.013 |
| Q99KQ4 | Nicotinamide phosphoribosyltransferase | 6 |  | 1.06 | 6 | 0.15 |  | 0.81 | 6 | 0.15 |  | 1.31 | 0.049 |
| Q9WU79 | Proline dehydrogenase 1, mitochondrial | 22 |  | 1.21 | 6 | 0.18 |  | 0.93 | 6 | 0.33 |  | 1.30 | 0.046 |
| Q9JMH6 | Thioredoxin reductase 1, cytoplasmic | 7 |  | 1.16 | 6 | 0.22 |  | 0.89 | 5 | 0.13 |  | 1.30 | 0.037 |
| Q9DCS2 | UPF0585 protein C16orf13 homolog | 9 |  | 1.22 | 6 | 0.26 |  | 0.95 | 6 | 0.21 |  | 1.29 | 0.031 |
| Q9JHK4 | Geranylgeranyl transferase type-2 subunit alpha | 2 |  | 1.00 | 6 | 0.09 |  | 0.78 | 5 | 0.21 |  | 1.29 | 0.033 |
| P14152 | Malate dehydrogenase, cytoplasmic | 26 |  | 1.26 | 6 | 0.29 |  | 0.97 | 6 | 0.09 |  | 1.29 | 0.033 |
| P09411 | Phosphoglycerate kinase 1 | 39 |  | 1.10 | 6 | 0.12 |  | 0.85 | 6 | 0.22 |  | 1.29 | 0.042 |
| Q9QUR6 | Prolyl endopeptidase | 13 |  | 0.97 | 6 | 0.07 |  | 0.75 | 5 | 0.25 |  | 1.29 | 0.032 |
| Q9QZW0 | Probable phospholipid-transporting ATPase 11C | 14 |  | 1.07 | 6 | 0.10 |  | 0.83 | 6 | 0.16 |  | 1.28 | 0.012 |
| Q9JMD3 | PCTP-like protein | 12 |  | 1.06 | 6 | 0.10 |  | 0.82 | 5 | 0.16 |  | 1.28 | 0.011 |
| P62827 | GTP-binding nuclear protein Ran | 6 |  | 1.03 | 6 | 0.07 |  | 0.81 | 5 | 0.25 |  | 1.27 | 0.045 |
| Q91XD4 | Formimidoyltransferase-cyclodeaminase | 41 |  | 1.25 | 6 | 0.16 |  | 0.98 | 6 | 0.15 |  | 1.27 | 0.042 |
| Q8QZR5 | Alanine aminotransferase 1 | 19 |  | 1.20 | 6 | 0.19 |  | 0.95 | 6 | 0.17 |  | 1.26 | 0.022 |
| Q91V64 | Isochorismatase domain-containing protein 1 | 5 |  | 1.12 | 6 | 0.18 |  | 0.90 | 5 | 0.05 |  | 1.25 | 0.026 |
| Q9QUH0 | Glutaredoxin-1 | 2 |  | 1.09 | 6 | 0.07 |  | 0.87 | 6 | 0.25 |  | 1.25 | 0.025 |
| P80316 | T-complex protein 1 subunit epsilon | 12 |  | 1.14 | 6 | 0.09 |  | 0.91 | 5 | 0.18 |  | 1.25 | 0.022 |
| P47962 | 60S ribosomal protein L5 | 13 |  | 1.16 | 6 | 0.23 |  | 0.92 | 5 | 0.04 |  | 1.25 | 0.044 |
| Q9DBF1 | Alpha-aminoadipic semialdehyde dehydrogenase | 36 |  | 1.18 | 6 | 0.17 |  | 0.95 | 6 | 0.06 |  | 1.24 | 0.005 |
| P17563 | Selenium-binding protein 1 | 126 |  | 1.15 | 6 | 0.12 |  | 0.94 | 6 | 0.16 |  | 1.23 | 0.031 |
| P47199 | Quinone oxidoreductase | 15 |  | 1.06 | 6 | 0.12 |  | 0.87 | 6 | 0.13 |  | 1.23 | 0.027 |
| P99029 | Peroxiredoxin-5, mitochondrial | 21 |  | 1.11 | 6 | 0.15 |  | 0.90 | 6 | 0.22 |  | 1.23 | 0.047 |
| P11725 | Ornithine carbamoyltransferase, mitochondrial | 63 |  | 1.13 | 6 | 0.10 |  | 0.92 | 6 | 0.10 |  | 1.23 | 0.036 |
| O88545 | COP9 signalosome complex subunit 6 | 2 |  | 1.05 | 6 | 0.14 |  | 0.86 | 5 | 0.09 |  | 1.22 | 0.046 |
| Q5YD48 | APOBEC1 complementation factor | 6 |  | 1.06 | 6 | 0.11 |  | 0.87 | 6 | 0.15 |  | 1.22 | 0.015 |
| O09172 | Glutamate--cysteine ligase regulatory subunit | 4 |  | 1.20 | 6 | 0.16 |  | 1.00 | 6 | 0.14 |  | 1.21 | 0.025 |
| P80314 | T-complex protein 1 subunit beta | 16 |  | 1.14 | 6 | 0.11 |  | 0.94 | 5 | 0.15 |  | 1.21 | 0.049 |
| Q60759 | Glutaryl-CoA dehydrogenase, mitochondrial | 23 |  | 1.15 | 6 | 0.16 |  | 0.95 | 6 | 0.09 |  | 1.21 | 0.033 |
| Q9D0F3 | Protein ERGIC-53 | 15 |  | 1.17 | 6 | 0.14 |  | 0.96 | 6 | 0.15 |  | 1.21 | 0.045 |
| Q9EQF5 | Dihydropyrimidinase | 20 |  | 1.15 | 6 | 0.12 |  | 0.95 | 5 | 0.13 |  | 1.20 | 0.044 |
| Q8K0Z7 | Translational activator of cytochrome c oxidase 1 | 3 |  | 0.99 | 6 | 0.08 |  | 1.16 | 6 | 0.06 |  | 0.85 | 0.024 |
| P10605 | Cathepsin B | 13 |  | 0.90 | 6 | 0.11 |  | 1.08 | 6 | 0.14 |  | 0.84 | 0.045 |
| Q9CY27 | Trans-2,3-enoyl-CoA reductase | 3 |  | 0.82 | 6 | 0.09 |  | 1.01 | 5 | 0.16 |  | 0.82 | 0.034 |
| Q8BWQ1 | UDP-glucuronosyltransferase 2A3 | 28 |  | 0.87 | 6 | 0.10 |  | 1.06 | 6 | 0.12 |  | 0.82 | 0.008 |
| Q9D8U8 | Sorting nexin-5 | 3 |  | 0.99 | 6 | 0.04 |  | 1.20 | 6 | 0.31 |  | 0.82 | 0.040 |
| Q8BG05 | Heterogeneous nuclear ribonucleoprotein A3 | 11 |  | 0.87 | 6 | 0.15 |  | 1.08 | 6 | 0.09 |  | 0.81 | 0.017 |
| P67984 | 60S ribosomal protein L22 | 5 |  | 0.98 | 6 | 0.14 |  | 1.23 | 6 | 0.16 |  | 0.80 | 0.005 |
| Q8BHN3 | Neutral alpha-glucosidase AB | 25 |  | 0.86 | 6 | 0.03 |  | 1.08 | 6 | 0.14 |  | 0.80 | 0.010 |
| Q9DD20 | Methyltransferase-like protein 7B | 15 |  | 0.89 | 6 | 0.17 |  | 1.11 | 5 | 0.21 |  | 0.80 | 0.044 |
| Q9JLT4 | Thioredoxin reductase 2, mitochondrial | 6 |  | 0.95 | 6 | 0.11 |  | 1.18 | 5 | 0.09 |  | 0.80 | 0.028 |
| O35129 | Prohibitin-2 | 21 |  | 0.97 | 6 | 0.03 |  | 1.20 | 6 | 0.17 |  | 0.80 | 0.009 |
| Q8CIN4 | Serine/threonine-protein kinase PAK 2 | 2 |  | 1.07 | 5 | 0.07 |  | 1.36 | 4 | 0.20 |  | 0.79 | 0.041 |
| B1AY13 | Ubiquitin carboxyl-terminal hydrolase 24 | 4 |  | 1.05 | 5 | 0.05 |  | 1.32 | 4 | 0.36 |  | 0.79 | 0.048 |
| Q61490 | CD166 antigen | 5 |  | 0.80 | 6 | 0.10 |  | 1.04 | 5 | 0.22 |  | 0.78 | 0.034 |
| P18242 | Cathepsin D | 11 |  | 0.84 | 6 | 0.15 |  | 1.08 | 6 | 0.19 |  | 0.78 | 0.029 |
| Q9EQH2 | Endoplasmic reticulum aminopeptidase 1 | 9 |  | 0.79 | 6 | 0.07 |  | 1.01 | 6 | 0.14 |  | 0.78 | 0.010 |
| Q9CQ80 | Vacuolar protein-sorting-associated protein 25 | 3 |  | 0.86 | 5 | 0.12 |  | 1.11 | 4 | 0.10 |  | 0.78 | 0.010 |
| Q91VT4 | Carbonyl reductase family member 4 | 4 |  | 1.10 | 6 | 0.24 |  | 1.41 | 5 | 0.30 |  | 0.78 | 0.040 |
| Q921X9 | Protein disulfide-isomerase A5 | 10 |  | 0.78 | 6 | 0.09 |  | 1.00 | 6 | 0.15 |  | 0.78 | 0.023 |
| Q8R0X7 | Sphingosine-1-phosphate lyase 1 | 3 |  | 0.93 | 5 | 0.09 |  | 1.19 | 4 | 0.09 |  | 0.78 | 0.014 |
| Q9DCN2 | NADH-cytochrome b5 reductase 3 | 27 |  | 0.96 | 6 | 0.10 |  | 1.25 | 6 | 0.21 |  | 0.77 | 0.010 |
| Q922J3 | CAP-Gly domain-containing linker protein 1 | 2 |  | 1.02 | 5 | 0.10 |  | 1.31 | 4 | 0.31 |  | 0.77 | 0.029 |
| P15327 | Bisphosphoglycerate mutase | 2 |  | 1.07 | 5 | 0.06 |  | 1.39 | 4 | 0.23 |  | 0.77 | 0.045 |
| Q9D0E1 | Heterogeneous nuclear ribonucleoprotein M | 9 |  | 0.93 | 6 | 0.22 |  | 1.23 | 6 | 0.27 |  | 0.76 | 0.022 |
| Q8VE38 | Oxidoreductase NAD-binding domain-containing protein 1 | 2 |  | 1.01 | 5 | 0.08 |  | 1.33 | 4 | 0.39 |  | 0.76 | 0.031 |
| Q8R0F8 | Acylpyruvase FAHD1, mitochondrial | 5 |  | 0.89 | 6 | 0.15 |  | 1.16 | 5 | 0.12 |  | 0.76 | 0.011 |
| Q60936 | Chaperone activity of bc1 complex-like, mitochondrial | 9 |  | 1.05 | 6 | 0.19 |  | 1.39 | 6 | 0.47 |  | 0.76 | 0.041 |
| Q9JLF6 | Protein-glutamine gamma-glutamyltransferase K | 5 |  | 0.95 | 6 | 0.20 |  | 1.24 | 5 | 0.23 |  | 0.76 | 0.049 |
| Q4LDG0 | Bile acyl-CoA synthetase | 30 |  | 0.80 | 6 | 0.17 |  | 1.05 | 6 | 0.12 |  | 0.76 | 0.026 |
| Q9ET22 | Dipeptidyl peptidase 2 | 2 |  | 0.88 | 6 | 0.32 |  | 1.18 | 6 | 0.27 |  | 0.75 | 0.043 |
| Q9DCX8 | Iodotyrosine dehalogenase 1 | 4 |  | 0.81 | 6 | 0.09 |  | 1.08 | 6 | 0.33 |  | 0.75 | 0.026 |
| O89017 | Legumain | 4 |  | 0.74 | 6 | 0.09 |  | 0.99 | 6 | 0.13 |  | 0.75 | 0.004 |
| P37040 | NADPH--cytochrome P450 reductase | 27 |  | 0.77 | 6 | 0.14 |  | 1.04 | 6 | 0.22 |  | 0.74 | 0.050 |
| Q7TNC4 | Putative RNA-binding protein Luc7-like 2 | 3 |  | 0.91 | 6 | 0.10 |  | 1.23 | 6 | 0.29 |  | 0.74 | 0.020 |
| Q99J39 | Malonyl-CoA decarboxylase, mitochondrial | 9 |  | 0.85 | 6 | 0.05 |  | 1.15 | 6 | 0.16 |  | 0.74 | 0.004 |
| P50427 | Steryl-sulfatase | 4 |  | 0.86 | 6 | 0.13 |  | 1.16 | 5 | 0.25 |  | 0.74 | 0.015 |
| Q9QXE0 | 2-hydroxyacyl-CoA lyase 1 | 33 |  | 0.91 | 6 | 0.20 |  | 1.26 | 6 | 0.21 |  | 0.73 | 0.001 |
| Q9DCT8 | Cysteine-rich protein 2 | 2 |  | 0.89 | 5 | 0.12 |  | 1.22 | 4 | 0.43 |  | 0.73 | 0.032 |
| P06683 | Complement component C9 | 3 |  | 0.84 | 6 | 0.16 |  | 1.16 | 5 | 0.09 |  | 0.73 | 0.014 |
| Q9CZU6 | Citrate synthase, mitochondrial | 23 |  | 0.85 | 6 | 0.09 |  | 1.17 | 6 | 0.22 |  | 0.73 | 0.023 |
| Q8BSY0 | Aspartyl/asparaginyl beta-hydroxylase | 4 |  | 0.94 | 6 | 0.19 |  | 1.29 | 5 | 0.31 |  | 0.73 | 0.037 |
| P97821 | Dipeptidyl peptidase 1 | 4 |  | 0.76 | 6 | 0.16 |  | 1.04 | 5 | 0.24 |  | 0.73 | 0.032 |
| P10833 | Ras-related protein R-Ras | 1 |  | 1.03 | 6 | 0.33 |  | 1.40 | 5 | 0.33 |  | 0.73 | 0.027 |
| P62806 | Histone H4 | 20 |  | 0.80 | 6 | 0.15 |  | 1.12 | 6 | 0.15 |  | 0.72 | 0.016 |
| Q9DC50 | Peroxisomal carnitine O-octanoyltransferase | 12 |  | 0.97 | 6 | 0.31 |  | 1.34 | 6 | 0.34 |  | 0.72 | 0.038 |
| Q9CYH2 | Redox-regulatory protein PAMM | 4 |  | 0.80 | 6 | 0.14 |  | 1.11 | 6 | 0.12 |  | 0.72 | 0.003 |
| Q02248 | Catenin beta-1 | 11 |  | 1.01 | 6 | 0.25 |  | 1.43 | 6 | 0.38 |  | 0.71 | 0.020 |
| Q80X19 | Collagen alpha-1(XIV) chain | 4 |  | 1.15 | 5 | 0.30 |  | 1.61 | 4 | 0.34 |  | 0.71 | 0.005 |
| Q8C854 | Myelin expression factor 2 | 1 |  | 0.88 | 5 | 0.09 |  | 1.26 | 4 | 0.10 |  | 0.70 | 0.002 |
| Q811U4 | Mitofusin-1 | 2 |  | 1.02 | 6 | 0.14 |  | 1.45 | 6 | 0.55 |  | 0.70 | 0.034 |
| Q9D2G2 | Dihydrolipoyllysine-residue succinyltransferase component of 2-oxoglutarate dehydrogenase complex, mitochondrial | 14 |  | 0.85 | 6 | 0.13 |  | 1.21 | 6 | 0.36 |  | 0.70 | 0.049 |
| Q64FW2 | All-trans-retinol 13,14-reductase | 8 |  | 0.93 | 6 | 0.29 |  | 1.34 | 6 | 0.54 |  | 0.69 | 0.041 |
| P48678 | Prelamin-A/C | 18 |  | 0.71 | 6 | 0.20 |  | 1.05 | 6 | 0.10 |  | 0.68 | 0.002 |
| Q4VBD2 | Transmembrane anterior posterior transformation protein 1 | 1 |  | 1.00 | 5 | 0.06 |  | 1.46 | 4 | 0.52 |  | 0.68 | 0.012 |
| P25688 | Uricase | 36 |  | 0.82 | 6 | 0.28 |  | 1.24 | 6 | 0.35 |  | 0.67 | 0.027 |
| Q8VEH5 | EPM2A-interacting protein 1 | 2 |  | 0.86 | 5 | 0.09 |  | 1.28 | 4 | 0.44 |  | 0.67 | 0.019 |
| P08032 | Spectrin alpha chain, erythrocyte | 7 |  | 0.96 | 5 | 0.18 |  | 1.45 | 4 | 0.34 |  | 0.66 | 0.005 |
| P21981 | Protein-glutamine gamma-glutamyltransferase 2 | 16 |  | 0.74 | 6 | 0.12 |  | 1.12 | 6 | 0.26 |  | 0.66 | 0.049 |
| Q9WU19 | Hydroxyacid oxidase 1 | 9 |  | 0.76 | 6 | 0.10 |  | 1.16 | 6 | 0.14 |  | 0.65 | <0.001 |
| Q6ZWY9 | Histone H2B type 1-C/E/G | 30 |  | 0.69 | 6 | 0.23 |  | 1.06 | 5 | 0.30 |  | 0.65 | 0.020 |
| O08917 | Flotillin-1 | 2 |  | 1.05 | 6 | 0.34 |  | 1.68 | 5 | 0.37 |  | 0.63 | 0.002 |
| P32020 | Non-specific lipid-transfer protein | 72 |  | 0.66 | 6 | 0.18 |  | 1.08 | 6 | 0.33 |  | 0.62 | 0.003 |
| Q99P30 | Peroxisomal coenzyme A diphosphatase NUDT7 | 28 |  | 0.72 | 6 | 0.15 |  | 1.16 | 6 | 0.41 |  | 0.62 | 0.008 |
| Q9CQC9 | GTP-binding protein SAR1b | 15 |  | 1.03 | 6 | 0.36 |  | 1.72 | 6 | 0.60 |  | 0.60 | 0.010 |
| P11714 | Cytochrome P450 2D9 | 42 |  | 0.83 | 6 | 0.12 |  | 1.43 | 6 | 0.44 |  | 0.58 | 0.008 |
| Q05816 | Fatty acid-binding protein, epidermal | 8 |  | 0.51 | 6 | 0.17 |  | 1.25 | 5 | 0.54 |  | 0.40 | 0.003 |
| O35728 | Cytochrome P450 4A14 | 7 |  | 0.66 | 6 | 0.33 |  | 1.70 | 6 | 1.29 |  | 0.39 | 0.018 |
