# Supplementary material for: Identification and quantification of the basal and inducible Nrf2-dependent proteomes in mouse liver: Biochemical, pharmacological and toxicological implications
Source: J Proteomics. 2014 Aug 28;108(100):171–87. doi: 10.1016/j.jprot.2014.05.007 (PMC4115266; doi:10.1016/j.jprot.2014.05.007)
Supplement: Supplementary Table 2 — iTRAQ-based proteomic comparison of liver proteins in vehicle treated and CDDO-me treated wild type mice. Proteins listed are those whose expression was different (P < 0.05) between wild type (Nrf2(+/+)) and wild type CDDO-me (Nrf2(+/+)CDDO) treated mice. Mean expression values relative to a common pool are given for n = 4–6 animals. Proteins are ordered according to the ratio between CDDO-me treated wild type mice and vehicle control treated wild type mice (Nrf2(+/+)CDDO/Nrf2(+/+); highest to lowest) such that proteins whose expression is most markedly induced by CDDO-me appear at the top of the list. aAverage number of peptides used for quantification across the four individual iTRAQ runs. [file mmc2.docx]

**Supplementary Table 2:**  *iTRAQ-based proteomic comparison of liver proteins in vehicle treated and CDDO-me treated wild type mice.* Proteins listed are those whose expression was different (P < 0.05) between wild type (Nrf2^(+/+)^) and wild type CDDO-me (Nrf2^(+/+)^CDDO) treated mice. Mean expression values relative to a common pool are given for n=4-6 animals. Proteins are ordered according to the ratio between CDDO-me treated wild type mice and vehicle control treated wild type mice (Nrf2^(+/+)^CDDO/Nrf2^(+/+)^) highest to lowest such that proteins whose expression is most markedly induced by CDDO-me appear at the top of the list.

*^a^*Average number of peptides used for quantification across the four individual iTRAQ runs.

| **Uniprot** |  |  |  | **Nrf2^(+/+)^** | | |  | **Nrf2^(+/+)^ CDDO** | | |  | **Nrf2^(+/+)^CDDO Nrf2^(+/+)^** |  |
| --- | --- | --- | --- | --- | --- | --- | --- | --- | --- | --- | --- | --- | --- |
| **Accession** | **Name** | **Peptides*^a^*** |  | **mean** | **n** | **SD** |  | **mean** | **n** | **SD** |  | **ratio** | **P-value** |
| P20852 | Cytochrome P450 2A5 | 11 |  | 0.40 | 6 | 0.10 |  | 3.26 | 6 | 1.03 |  | 8.12 | <0.001 |
| Q9WUZ9 | Ectonucleoside triphosphate diphosphohydrolase 5 | 8 |  | 1.05 | 6 | 0.19 |  | 2.15 | 6 | 0.73 |  | 2.04 | <0.001 |
| Q8JZK9 | Hydroxymethylglutaryl-CoA synthase, cytoplasmic | 10 |  | 0.72 | 6 | 0.11 |  | 1.26 | 5 | 0.29 |  | 1.76 | 0.016 |
| Q8C165 | Probable carboxypeptidase PM20D1 | 4 |  | 0.73 | 6 | 0.42 |  | 1.20 | 5 | 0.29 |  | 1.64 | 0.049 |
| P48758 | Carbonyl reductase [NADPH] 1 | 18 |  | 0.87 | 6 | 0.11 |  | 1.42 | 6 | 0.33 |  | 1.63 | <0.001 |
| P19639 | Glutathione S-transferase Mu 3 | 49 |  | 1.33 | 6 | 0.19 |  | 2.11 | 6 | 0.59 |  | 1.58 | <0.001 |
| O70475 | UDP-glucose 6-dehydrogenase | 24 |  | 1.19 | 6 | 0.36 |  | 1.87 | 6 | 0.40 |  | 1.57 | <0.001 |
| Q9QYF1 | Retinol dehydrogenase 11 | 2 |  | 0.86 | 5 | 0.18 |  | 1.31 | 4 | 0.20 |  | 1.52 | 0.042 |
| P58044 | Isopentenyl-diphosphate Delta-isomerase 1 | 3 |  | 0.84 | 6 | 0.08 |  | 1.23 | 6 | 0.41 |  | 1.48 | 0.037 |
| Q9D379 | Epoxide hydrolase 1 | 14 |  | 1.25 | 6 | 0.37 |  | 1.86 | 6 | 0.48 |  | 1.48 | 0.002 |
| P50285 | Dimethylaniline monooxygenase [N-oxide-forming] 1 | 18 |  | 1.03 | 6 | 0.08 |  | 1.50 | 4 | 0.33 |  | 1.45 | 0.001 |
| P10649 | Glutathione S-transferase Mu 1 | 69 |  | 1.03 | 6 | 0.29 |  | 1.47 | 6 | 0.26 |  | 1.43 | 0.022 |
| Q9R1J0 | Sterol-4-alpha-carboxylate 3-dehydrogenase, decarboxylating | 8 |  | 0.77 | 6 | 0.10 |  | 1.09 | 6 | 0.27 |  | 1.42 | 0.034 |
| Q07076 | Annexin A7 | 4 |  | 0.94 | 6 | 0.11 |  | 1.34 | 6 | 0.27 |  | 1.42 | 0.003 |
| P38060 | Hydroxymethylglutaryl-CoA lyase, mitochondrial | 21 |  | 0.85 | 6 | 0.25 |  | 1.14 | 6 | 0.26 |  | 1.34 | 0.017 |
| Q9DD20 | Methyltransferase-like protein 7B | 15 |  | 0.89 | 6 | 0.17 |  | 1.18 | 4 | 0.08 |  | 1.33 | 0.015 |
| Q923D2 | Flavin reductase (NADPH) | 9 |  | 1.30 | 6 | 0.09 |  | 1.73 | 6 | 0.35 |  | 1.33 | 0.004 |
| P29341 | Polyadenylate-binding protein 1 | 18 |  | 0.93 | 6 | 0.10 |  | 1.23 | 6 | 0.16 |  | 1.32 | 0.014 |
| E9Q557 | Desmoplakin | 5 |  | 0.84 | 6 | 0.10 |  | 1.08 | 6 | 0.25 |  | 1.29 | 0.027 |
| P62900 | 60S ribosomal protein L31 | 6 |  | 0.92 | 6 | 0.14 |  | 1.19 | 6 | 0.27 |  | 1.29 | 0.038 |
| Q9QXD1 | Peroxisomal acyl-coenzyme A oxidase 2 | 10 |  | 0.98 | 6 | 0.15 |  | 1.27 | 6 | 0.19 |  | 1.29 | 0.017 |
| Q61233 | Plastin-2 | 19 |  | 1.02 | 5 | 0.21 |  | 1.31 | 4 | 0.33 |  | 1.28 | 0.026 |
| Q9EQH2 | Endoplasmic reticulum aminopeptidase 1 | 9 |  | 0.79 | 6 | 0.07 |  | 0.99 | 6 | 0.10 |  | 1.26 | 0.016 |
| Q99J39 | Malonyl-CoA decarboxylase, mitochondrial | 9 |  | 0.85 | 6 | 0.05 |  | 1.06 | 6 | 0.20 |  | 1.25 | 0.036 |
| O09172 | Glutamate--cysteine ligase regulatory subunit | 4 |  | 1.20 | 6 | 0.16 |  | 1.40 | 6 | 0.26 |  | 1.17 | 0.030 |
| Q8BGC4 | Zinc-binding alcohol dehydrogenase domain-containing protein 2 | 8 |  | 0.89 | 6 | 0.09 |  | 1.03 | 6 | 0.10 |  | 1.16 | 0.049 |
| P62983 | Ubiquitin-40S ribosomal protein S27a | 17 |  | 1.03 | 5 | 0.10 |  | 0.84 | 5 | 0.27 |  | 0.82 | 0.047 |
| Q99KI0 | Aconitate hydratase, mitochondrial | 42 |  | 1.16 | 6 | 0.14 |  | 0.95 | 6 | 0.15 |  | 0.82 | 0.041 |
| Q921F4 | Heterogeneous nuclear ribonucleoprotein L-like | 2 |  | 1.19 | 6 | 0.19 |  | 0.95 | 4 | 0.06 |  | 0.81 | 0.049 |
| Q9QZW0 | Probable phospholipid-transporting ATPase 11C | 14 |  | 1.07 | 6 | 0.10 |  | 0.86 | 6 | 0.15 |  | 0.81 | 0.025 |
| Q8VDM4 | 26S proteasome non-ATPase regulatory subunit 2 | 18 |  | 1.15 | 6 | 0.20 |  | 0.93 | 6 | 0.08 |  | 0.81 | 0.043 |
| Q9DCW4 | Electron transfer flavoprotein subunit beta | 33 |  | 1.03 | 6 | 0.07 |  | 0.82 | 6 | 0.18 |  | 0.80 | 0.025 |
| O55234 | Proteasome subunit beta type-5 | 4 |  | 1.16 | 6 | 0.07 |  | 0.92 | 6 | 0.26 |  | 0.79 | 0.041 |
| Q64514 | Tripeptidyl-peptidase 2 | 10 |  | 1.32 | 6 | 0.12 |  | 1.02 | 6 | 0.25 |  | 0.77 | 0.034 |
| Q8C0Z1 | Protein ITFG3 | 3 |  | 1.14 | 5 | 0.11 |  | 0.86 | 5 | 0.25 |  | 0.76 | 0.040 |
| Q9DCM0 | Protein ETHE1, mitochondrial | 8 |  | 1.65 | 6 | 0.41 |  | 1.23 | 6 | 0.22 |  | 0.75 | 0.017 |
| Q91XD4 | Formimidoyltransferase-cyclodeaminase | 41 |  | 1.25 | 6 | 0.16 |  | 0.92 | 6 | 0.26 |  | 0.73 | 0.013 |
| Q922D8 | C-1-tetrahydrofolate synthase, cytoplasmic | 40 |  | 1.22 | 6 | 0.16 |  | 0.89 | 5 | 0.29 |  | 0.73 | 0.017 |
| Q9DAR7 | Scavenger mRNA-decapping enzyme DcpS | 4 |  | 1.09 | 6 | 0.15 |  | 0.78 | 6 | 0.47 |  | 0.72 | 0.029 |
| Q91Y97 | Fructose-bisphosphate aldolase B | 111 |  | 1.13 | 6 | 0.31 |  | 0.78 | 6 | 0.21 |  | 0.69 | 0.037 |
| Q6XVG2 | Cytochrome P450 2C54 | 16 |  | 1.41 | 6 | 0.29 |  | 0.95 | 5 | 0.29 |  | 0.67 | 0.009 |
| P70398 | Probable ubiquitin carboxyl-terminal hydrolase FAF-X | 7 |  | 1.07 | 5 | 0.11 |  | 0.69 | 5 | 0.24 |  | 0.64 | 0.011 |
| P70255 | Nuclear factor 1 C-type | 1 |  | 1.27 | 5 | 0.27 |  | 0.78 | 5 | 0.28 |  | 0.61 | 0.012 |
