# Supplementary material for: Identification and quantification of the basal and inducible Nrf2-dependent proteomes in mouse liver: Biochemical, pharmacological and toxicological implications
Source: J Proteomics. 2014 Aug 28;108(100):171–87. doi: 10.1016/j.jprot.2014.05.007 (PMC4115266; doi:10.1016/j.jprot.2014.05.007)
Supplement: Supplementary Table 3 — iTRAQ-based proteomic comparison of liver proteins in vehicle control and CDDO-me treated Nrf2(−/−) mice. Proteins listed are those whose expression was different (P < 0.05) between Nrf2(−/−) and Nrf2(−/−)-CDDO-me treated mice. Mean expression values relative to a common pool are given for n = 4–6 animals. Proteins are ordered according to the ratio between CDDO-me treated Nrf2(−/−) mice and vehicle treated Nrf2(−/−) mice (Nrf2(−/−)CDDO/Nrf2(−/−); highest to lowest) such that proteins whose expression is most markedly induced by CDDO-me appear at the top of the list. aAverage number of peptides used for quantification across the four individual iTRAQ runs. [file mmc3.docx]

**Supplementary Table 3:**  *iTRAQ-based proteomic comparison of liver proteins in vehicle control and CDDO-me treated* Nrf2^(-/-)^ *mice.* Proteins listed are those whose expression was different (P < 0.05) between Nrf2^(-/-)^ and Nrf2^(-/-)^-CDDO-me treated mice. Mean expression values relative to a common pool are given for n=4-6 animals. Proteins are ordered according to the ratio between CDDO-me treated Nrf2^(-/-)^ mice and vehicle treated Nrf2^(-/-)^ mice (Nrf2^(-/-)^CDDO/Nrf2^(-/-)^) highest to lowest such that proteins whose expression is most markedly induced by CDDO-me appear at the top of the list. *^a^*Average number of peptides used for quantification across the four individual iTRAQ runs.

| **Uniprot** |  |  |  | **Nrf2^(-/-)^** | | |  | **Nrf2^(-/-)^ CDDO** | | |  | **Nrf2^(-/-)^ CDDO**  **Nrf2^(-/-)^** |  |
| --- | --- | --- | --- | --- | --- | --- | --- | --- | --- | --- | --- | --- | --- |
| **Accession** | **Name** | **Peptides*^a^*** |  | **mean** | **n** | **SD** |  | **mean** | **n** | **SD** |  | **ratio** | **P-value** |
| Q8C165 | Probable carboxypeptidase PM20D1 | 4 |  | 0.66 | 4 | 0.25 |  | 1.14 | 6 | 0.33 |  | 1.73 | 0.047 |
| O35386 | Phytanoyl-CoA dioxygenase, peroxisomal | 5 |  | 0.96 | 6 | 0.30 |  | 1.64 | 6 | 0.69 |  | 1.70 | 0.008 |
| P58044 | Isopentenyl-diphosphate Delta-isomerase 1 | 3 |  | 0.76 | 6 | 0.18 |  | 1.23 | 6 | 0.48 |  | 1.63 | 0.015 |
| P09103 | Protein disulfide-isomerase | 83 |  | 0.76 | 6 | 0.22 |  | 1.15 | 6 | 0.28 |  | 1.53 | 0.028 |
| Q8VCM7 | Fibrinogen gamma chain | 11 |  | 0.86 | 6 | 0.27 |  | 1.30 | 6 | 0.19 |  | 1.52 | <0.001 |
| P62082 | 40S ribosomal protein S7 | 17 |  | 0.77 | 6 | 0.20 |  | 1.15 | 6 | 0.24 |  | 1.49 | 0.013 |
| P19324 | Serpin H1 | 3 |  | 1.00 | 5 | 0.21 |  | 1.47 | 4 | 0.45 |  | 1.47 | 0.044 |
| Q9DBG7 | Signal recognition particle receptor subunit alpha | 4 |  | 0.81 | 5 | 0.20 |  | 1.20 | 4 | 0.18 |  | 1.47 | 0.019 |
| P24369 | Peptidyl-prolyl cis-trans isomerase B | 8 |  | 1.04 | 6 | 0.35 |  | 1.51 | 6 | 0.46 |  | 1.46 | 0.017 |
| Q8QZZ7 | TP53RK-binding protein | 1 |  | 0.81 | 5 | 0.25 |  | 1.18 | 4 | 0.19 |  | 1.46 | 0.042 |
| P27773 | Protein disulfide-isomerase A3 | 49 |  | 0.93 | 6 | 0.21 |  | 1.34 | 6 | 0.30 |  | 1.44 | 0.016 |
| O08600 | Endonuclease G, mitochondrial | 3 |  | 0.99 | 5 | 0.05 |  | 1.39 | 4 | 0.30 |  | 1.40 | 0.004 |
| Q9JHK4 | Geranylgeranyl transferase type-2 subunit alpha | 2 |  | 0.78 | 5 | 0.21 |  | 1.09 | 4 | 0.18 |  | 1.40 | 0.010 |
| P18760 | Cofilin-1 | 14 |  | 0.81 | 6 | 0.18 |  | 1.12 | 6 | 0.23 |  | 1.39 | 0.013 |
| Q8BW75 | Amine oxidase [flavin-containing] B | 18 |  | 1.04 | 6 | 0.21 |  | 1.42 | 6 | 0.36 |  | 1.37 | 0.013 |
| P49722 | Proteasome subunit alpha type-2 | 11 |  | 0.95 | 6 | 0.17 |  | 1.30 | 6 | 0.37 |  | 1.37 | 0.024 |
| Q922E4 | Ethanolamine-phosphate cytidylyltransferase | 9 |  | 0.93 | 6 | 0.17 |  | 1.27 | 6 | 0.34 |  | 1.37 | 0.012 |
| P99027 | 60S acidic ribosomal protein P2 | 19 |  | 0.98 | 6 | 0.12 |  | 1.32 | 6 | 0.23 |  | 1.36 | 0.020 |
| P14211 | Calreticulin | 24 |  | 0.90 | 6 | 0.29 |  | 1.23 | 6 | 0.23 |  | 1.36 | 0.038 |
| Q9CQF9 | Prenylcysteine oxidase | 5 |  | 0.72 | 5 | 0.21 |  | 0.98 | 4 | 0.05 |  | 1.36 | 0.036 |
| O08795 | Glucosidase 2 subunit beta | 6 |  | 1.06 | 6 | 0.18 |  | 1.43 | 6 | 0.15 |  | 1.34 | 0.001 |
| Q921M3 | Splicing factor 3B subunit 3 | 4 |  | 0.82 | 5 | 0.15 |  | 1.10 | 4 | 0.16 |  | 1.34 | 0.009 |
| Q9DCM2 | Glutathione S-transferase kappa 1 | 9 |  | 1.01 | 6 | 0.08 |  | 1.34 | 6 | 0.40 |  | 1.33 | 0.040 |
| O70503 | Estradiol 17-beta-dehydrogenase 12 | 6 |  | 0.91 | 6 | 0.17 |  | 1.20 | 6 | 0.19 |  | 1.33 | 0.032 |
| P62702 | 40S ribosomal protein S4, X isoform | 13 |  | 0.74 | 6 | 0.21 |  | 0.99 | 6 | 0.12 |  | 1.33 | 0.043 |
| P47962 | 60S ribosomal protein L5 | 13 |  | 0.92 | 5 | 0.04 |  | 1.22 | 4 | 0.14 |  | 1.32 | 0.024 |
| Q60866 | Phosphotriesterase-related protein | 7 |  | 1.00 | 6 | 0.15 |  | 1.31 | 6 | 0.24 |  | 1.31 | 0.006 |
| Q9CXI5 | Mesencephalic astrocyte-derived neurotrophic factor | 4 |  | 0.89 | 5 | 0.23 |  | 1.16 | 4 | 0.16 |  | 1.30 | 0.033 |
| P62827 | GTP-binding nuclear protein Ran | 6 |  | 0.81 | 5 | 0.25 |  | 1.06 | 4 | 0.12 |  | 1.30 | 0.042 |
| Q8K0C4 | Lanosterol 14-alpha demethylase | 5 |  | 0.90 | 6 | 0.16 |  | 1.16 | 6 | 0.28 |  | 1.29 | 0.031 |
| Q8BHC4 | Dephospho-CoA kinase domain-containing protein | 2 |  | 0.77 | 5 | 0.19 |  | 0.99 | 4 | 0.06 |  | 1.29 | 0.028 |
| Q5XJY5 | Coatomer subunit delta | 7 |  | 0.97 | 6 | 0.07 |  | 1.24 | 6 | 0.29 |  | 1.28 | 0.042 |
| P29341 | Polyadenylate-binding protein 1 | 18 |  | 0.94 | 6 | 0.18 |  | 1.20 | 6 | 0.29 |  | 1.28 | 0.028 |
| P62806 | Histone H4 | 20 |  | 1.12 | 6 | 0.15 |  | 1.42 | 6 | 0.34 |  | 1.27 | 0.020 |
| P05202 | Aspartate aminotransferase, mitochondrial | 71 |  | 1.02 | 6 | 0.10 |  | 1.27 | 6 | 0.16 |  | 1.25 | 0.044 |
| O35887 | Calumenin | 8 |  | 0.99 | 4 | 0.20 |  | 1.23 | 6 | 0.09 |  | 1.25 | 0.014 |
| P97461 | 40S ribosomal protein S5 | 9 |  | 0.83 | 5 | 0.14 |  | 1.03 | 4 | 0.09 |  | 1.25 | 0.039 |
| Q8C0C7 | Phenylalanyl-tRNA synthetase alpha chain | 5 |  | 0.78 | 6 | 0.14 |  | 0.98 | 6 | 0.05 |  | 1.25 | 0.018 |
| P57780 | Alpha-actinin-4 | 42 |  | 1.10 | 6 | 0.11 |  | 1.35 | 6 | 0.25 |  | 1.23 | 0.010 |
| P26516 | 26S proteasome non-ATPase regulatory subunit 7 | 10 |  | 0.95 | 4 | 0.26 |  | 1.16 | 6 | 0.12 |  | 1.22 | 0.049 |
| Q60864 | Stress-induced-phosphoprotein 1 | 9 |  | 0.88 | 6 | 0.06 |  | 1.06 | 6 | 0.12 |  | 1.21 | 0.008 |
| P28656 | Nucleosome assembly protein 1-like 1 | 4 |  | 0.83 | 4 | 0.13 |  | 0.98 | 6 | 0.05 |  | 1.18 | 0.038 |
| Q6P5E4 | UDP-glucose:glycoprotein glucosyltransferase 1 | 18 |  | 1.01 | 6 | 0.13 |  | 1.18 | 6 | 0.17 |  | 1.17 | 0.043 |
| Q8K0Z7 | Translational activator of cytochrome c oxidase 1 | 3 |  | 1.16 | 6 | 0.06 |  | 0.99 | 6 | 0.14 |  | 0.85 | 0.025 |
| P67984 | 60S ribosomal protein L22 | 5 |  | 1.23 | 6 | 0.16 |  | 1.03 | 6 | 0.11 |  | 0.84 | 0.023 |
| Q60931 | Voltage-dependent anion-selective channel protein 3 | 7 |  | 1.19 | 6 | 0.26 |  | 0.99 | 6 | 0.06 |  | 0.84 | 0.037 |
| Q9Z1Q9 | Valyl-tRNA synthetase | 18 |  | 1.12 | 6 | 0.15 |  | 0.92 | 6 | 0.19 |  | 0.82 | 0.030 |
| Q9EP69 | Phosphatidylinositide phosphatase SAC1 | 6 |  | 1.07 | 6 | 0.23 |  | 0.88 | 6 | 0.15 |  | 0.82 | 0.047 |
| Q9DCD0 | 6-phosphogluconate dehydrogenase, decarboxylating | 8 |  | 1.14 | 6 | 0.14 |  | 0.92 | 6 | 0.21 |  | 0.81 | 0.043 |
| Q9EQU5 | Protein SET | 6 |  | 1.11 | 6 | 0.14 |  | 0.90 | 6 | 0.13 |  | 0.81 | 0.027 |
| Q922J3 | CAP-Gly domain-containing linker protein 1 | 2 |  | 1.31 | 4 | 0.31 |  | 1.05 | 6 | 0.21 |  | 0.80 | 0.046 |
| Q5XG73 | Acyl-CoA-binding domain-containing protein 5 | 4 |  | 1.27 | 6 | 0.26 |  | 1.01 | 6 | 0.12 |  | 0.80 | 0.032 |
| Q80X19 | Collagen alpha-1(XIV) chain | 4 |  | 1.61 | 4 | 0.34 |  | 1.27 | 6 | 0.14 |  | 0.79 | 0.028 |
| P52825 | Carnitine O-palmitoyltransferase 2, mitochondrial | 21 |  | 1.26 | 6 | 0.24 |  | 1.00 | 6 | 0.22 |  | 0.79 | 0.045 |
| Q8VCB3 | Glycogen [starch] synthase, liver | 10 |  | 1.18 | 6 | 0.21 |  | 0.92 | 6 | 0.21 |  | 0.79 | 0.012 |
| P47754 | F-actin-capping protein subunit alpha-2 | 6 |  | 1.14 | 6 | 0.15 |  | 0.90 | 6 | 0.15 |  | 0.79 | 0.011 |
| Q9JLT4 | Thioredoxin reductase 2, mitochondrial | 6 |  | 1.18 | 5 | 0.09 |  | 0.91 | 4 | 0.17 |  | 0.77 | 0.021 |
| Q3U2P1 | Protein transport protein Sec24A | 11 |  | 1.16 | 6 | 0.16 |  | 0.89 | 6 | 0.16 |  | 0.77 | 0.010 |
| Q8BVE3 | V-type proton ATPase subunit H | 3 |  | 1.39 | 5 | 0.41 |  | 1.06 | 4 | 0.11 |  | 0.76 | 0.043 |
| Q91VT4 | Carbonyl reductase family member 4 | 4 |  | 1.41 | 5 | 0.30 |  | 1.06 | 4 | 0.10 |  | 0.75 | 0.035 |
| Q60936 | Chaperone activity of bc1 complex-like, mitochondrial | 9 |  | 1.39 | 6 | 0.47 |  | 1.04 | 6 | 0.19 |  | 0.75 | 0.032 |
| P97855 | Ras GTPase-activating protein-binding protein 1 | 6 |  | 1.30 | 5 | 0.31 |  | 0.97 | 4 | 0.07 |  | 0.75 | 0.029 |
| Q8C854 | Myelin expression factor 2 | 1 |  | 1.26 | 4 | 0.10 |  | 0.95 | 6 | 0.20 |  | 0.75 | 0.006 |
| Q80Y14 | Glutaredoxin-related protein 5, mitochondrial | 3 |  | 1.22 | 4 | 0.37 |  | 0.91 | 6 | 0.23 |  | 0.75 | 0.045 |
| Q9CQZ5 | NADH dehydrogenase [ubiquinone] 1 alpha subcomplex subunit 6 | 4 |  | 1.17 | 6 | 0.23 |  | 0.88 | 6 | 0.21 |  | 0.75 | 0.014 |
| Q78IK4 | Apolipoprotein O-like | 4 |  | 1.07 | 4 | 0.07 |  | 0.81 | 6 | 0.15 |  | 0.75 | 0.005 |
| P15327 | Bisphosphoglycerate mutase | 2 |  | 1.39 | 4 | 0.23 |  | 1.03 | 6 | 0.28 |  | 0.74 | 0.020 |
| Q9CXF0 | Kynureninase | 11 |  | 1.02 | 6 | 0.16 |  | 0.76 | 6 | 0.26 |  | 0.74 | 0.032 |
| Q8VE37 | Regulator of chromosome condensation | 1 |  | 1.33 | 4 | 0.37 |  | 0.97 | 6 | 0.11 |  | 0.73 | 0.034 |
| Q8VEH5 | EPM2A-interacting protein 1 | 2 |  | 1.28 | 4 | 0.44 |  | 0.93 | 6 | 0.18 |  | 0.73 | 0.038 |
| Q05421 | Cytochrome P450 2E1 | 33 |  | 1.05 | 6 | 0.26 |  | 0.76 | 6 | 0.31 |  | 0.73 | 0.046 |
| P08032 | Spectrin alpha chain, erythrocyte | 7 |  | 1.45 | 4 | 0.34 |  | 1.04 | 6 | 0.19 |  | 0.72 | 0.014 |
| P42125 | Enoyl-CoA delta isomerase 1, mitochondrial | 13 |  | 1.05 | 6 | 0.31 |  | 0.75 | 6 | 0.17 |  | 0.71 | 0.029 |
| Q8VI47 | Canalicular multispecific organic anion transporter 1 | 12 |  | 0.97 | 6 | 0.16 |  | 0.69 | 6 | 0.37 |  | 0.71 | 0.049 |
| Q9Z0M5 | Lysosomal acid lipase/cholesteryl ester hydrolase | 5 |  | 1.27 | 4 | 0.19 |  | 0.88 | 6 | 0.28 |  | 0.70 | 0.031 |
| O08917 | Flotillin-1 | 2 |  | 1.68 | 5 | 0.37 |  | 1.16 | 4 | 0.34 |  | 0.69 | 0.018 |
| Q99L13 | 3-hydroxyisobutyrate dehydrogenase, mitochondrial | 14 |  | 1.39 | 6 | 0.52 |  | 0.96 | 6 | 0.15 |  | 0.69 | 0.041 |
| P55050 | Fatty acid-binding protein, intestinal | 2 |  | 1.31 | 5 | 0.36 |  | 0.91 | 4 | 0.10 |  | 0.69 | 0.028 |
| Q62189 | U1 small nuclear ribonucleoprotein A | 1 |  | 1.30 | 5 | 0.45 |  | 0.88 | 4 | 0.17 |  | 0.67 | 0.017 |
| P61924 | Coatomer subunit zeta-1 | 1 |  | 1.26 | 6 | 0.31 |  | 0.85 | 6 | 0.33 |  | 0.67 | 0.040 |
| Q9CQC9 | GTP-binding protein SAR1b | 15 |  | 1.72 | 6 | 0.60 |  | 1.09 | 6 | 0.23 |  | 0.63 | 0.017 |
| Q4VBD2 | Transmembrane anterior posterior transformation protein 1 | 1 |  | 1.46 | 4 | 0.52 |  | 0.89 | 6 | 0.11 |  | 0.61 | 0.002 |
| Q91WL5 | Cytochrome P450 4A12A | 17 |  | 1.15 | 6 | 0.40 |  | 0.69 | 6 | 0.30 |  | 0.60 | 0.024 |
| Q3ULD5 | Methylcrotonoyl-CoA carboxylase beta chain, mitochondrial | 14 |  | 1.31 | 6 | 0.23 |  | 0.76 | 6 | 0.27 |  | 0.58 | <0.001 |
| Q9DCY0 | Glycine N-acyltransferase-like protein Keg1 | 12 |  | 1.07 | 6 | 0.26 |  | 0.60 | 6 | 0.16 |  | 0.56 | 0.021 |
| Q64FW2 | All-trans-retinol 13,14-reductase | 8 |  | 1.34 | 6 | 0.54 |  | 0.74 | 6 | 0.18 |  | 0.55 | 0.004 |
| O09158 | Cytochrome P450 3A25 | 4 |  | 1.44 | 5 | 0.60 |  | 0.78 | 4 | 0.25 |  | 0.54 | 0.032 |
| Q99LY9 | NADH dehydrogenase [ubiquinone] iron-sulfur protein 5 | 2 |  | 1.78 | 5 | 1.25 |  | 0.82 | 4 | 0.28 |  | 0.46 | 0.031 |
| Q64459 | Cytochrome P450 3A11 | 27 |  | 1.38 | 6 | 0.34 |  | 0.63 | 6 | 0.26 |  | 0.46 | <0.001 |
| O35728 | Cytochrome P450 4A14 | 7 |  | 1.70 | 6 | 1.29 |  | 0.71 | 6 | 0.39 |  | 0.42 | 0.023 |
| O88833 | Cytochrome P450 4A10 | 9 |  | 1.92 | 4 | 1.69 |  | 0.55 | 6 | 0.48 |  | 0.29 | 0.012 |
