# Supplementary material for: Identification and quantification of the basal and inducible Nrf2-dependent proteomes in mouse liver: Biochemical, pharmacological and toxicological implications
Source: J Proteomics. 2014 Aug 28;108(100):171–87. doi: 10.1016/j.jprot.2014.05.007 (PMC4115266; doi:10.1016/j.jprot.2014.05.007)
Supplement: Supplementary Table 4 — Enhancer element binding site analysis of the proteins regulated by Nrf2 at both the constitutive and inducible levels. Full length gene and promoter (3000 bp) DNA sequences were interrogated for consensus Nrf2 binding sites using the MatInspector search in the Genomatix software suite, focussing on the MAF and AP1 related factor subgroup (V$AP1R). Full length gene sequences were retrieved from Entrez gene, while promoter sequences were extracted using the Gene2Promoter tool of the Genomatix software. Matrix similarity was optimised and core similarity was set to 0.75. Results presented in the table are limited to sequences associated with three transcription factor binding sites: NF-E2 p45, antioxidant response elements and binding sites for heterodimers with small Maf-proteins. [file mmc4.docx]

**Supplementary Table 4:**  *Enhancer element binding site analysis of the proteins regulated by Nrf2 at both the constitutive and inducible levels.* Full length gene and promoter (3000bp) DNA sequences were interrogated for consensus Nrf2 binding sites using the MatInspector search in the Genomatix software suite, focussing on the MAF and AP1 related factor subgroup (V$AP1R). Full length gene sequences were retrieved from Entrez gene, while promoter sequences were extracted using the Gene2Promoter tool of the Genomatix software. Matrix similarity was optimised and core similarity was set to 0.75. Results presented in the table are limited to sequences associated with three transcription factor binding sites: NF-E2 p45, antioxidant response elements and binding sites for heterodimers with small Maf-proteins.

| **Gene** | **Uniprot accession number** | **Transcription factor binding site** | **Matrix similarity** | | **Strand** | **Sequence** |
| --- | --- | --- | --- | --- | --- | --- |
|  |  |  | All positions | Core positions |  |  |
| ***Entpd5*** | Q9WUZ9 |  |  |  |  |  |
| Promoter |  | NF-E2 p45 | 0.94 | 1 | - | tactgCTGAttcacctgaggc |
|  |  | NF-E2 p45 | 0.866 | 0.774 | - | aagagGTGActcatgggaaat |
|  |  | Binding sites for heterodimers with small Maf-proteins | 0.82 | 0.81 | + | cactGCTTggtcaaggatttc |
|  |  | NF-E2 p45 | 0.866 | 1 | + | aaccgCTGAgccatctctcca |
|  |  | Binding sites for heterodimers with small Maf-proteins | 0.894 | 0.776 | - | ttttGCTAactcattctcttc |
|  |  | NF-E2 p45 | 0.855 | 0.774 | + | acaggGTGActcatttgtgaa |
|  |  | Antioxidant response elements | 0.82 | 0.75 | - | acaatgAGACaaagcaaattc |
|  |  | NF-E2 p45 | 0.855 | 0.774 | - | taaggGTGActcatctaactg |
| Full length gene |  | Antioxidant response elements | 0.765 | 1 | - | taaaaaTGACtcacctcaaac |
|  |  | Binding sites for heterodimers with small Maf-proteins | 0.844 | 1 | + | aactGCTGcttcagtttttct |
|  |  | NF-E2 p45 | 0.877 | 1 | + | aactgCTGAgccatctctcca |
|  |  | Antioxidant response elements | 0.822 | 1 | - | tgagccTGACtgagctacaat |
|  |  | Binding sites for heterodimers with small Maf-proteins | 0.851 | 0.776 | + | tgtaGCTCagtcaggctcaaa |
|  |  | Binding sites for heterodimers with small Maf-proteins | 0.862 | 1 | - | cagaGCTGtttcatccccgtg |
|  |  | NF-E2 p45 | 0.857 | 0.774 | + | gggtgGTGAttcatacctgta |
|  |  | NF-E2 p45 | 0.866 | 1 | + | gaaagCTGAgtaacccacact |
|  |  | Binding sites for heterodimers with small Maf-proteins | 0.842 | 0.776 | + | gatgGCTCagtcaggaaggcg |
|  |  | Antioxidant response elements | 0.783 | 1 | - | cccccgTGACccagcttcctg |
|  |  | Antioxidant response elements | 0.78 | 1 | + | acatcaTGACcaagaagcaag |
|  |  | Antioxidant response elements | 0.825 | 1 | - | tcaactTGACacagctggagt |
|  |  | Binding sites for heterodimers with small Maf-proteins | 0.87 | 1 | + | ccctGCTGtttcagagctccg |
|  |  | Antioxidant response elements | 0.765 | 1 | + | ttactgTGACtgatctatgtg |
|  |  | Antioxidant response elements | 0.773 | 1 | - | ttacaaTGACtgcccagcatc |
|  |  | NF-E2 p45 | 0.9 | 1 | - | tggttCTGActcattctctca |
|  |  | NF-E2 p45 | 0.88 | 1 | - | gtcttCTGAatcacaggtcag |
|  |  | Binding sites for heterodimers with small Maf-proteins | 0.833 | 1 | - | cagtGCTGactgacactggat |
|  |  | Antioxidant response elements | 0.761 | 1 | - | ttatctTGACagagtaagaat |
|  |  | Antioxidant response elements | 0.769 | 0.75 | + | gcaaacTGGCtcagcagggaa |
|  |  | Binding sites for heterodimers with small Maf-proteins | 0.831 | 1 | + | gtggGCTGcatcactaagaac |
|  |  | Binding sites for heterodimers with small Maf-proteins | 0.851 | 1 | + | gagaGCTGtgtcaaaagaaga |
|  |  | Antioxidant response elements | 0.783 | 1 | + | taaaggTGACtcagatggcta |
|  |  | Binding sites for heterodimers with small Maf-proteins | 0.831 | 1 | - | cctaGCTGtctcaagaccctg |
| ***Cyp2a5*** | P20852 |  |  |  |  |  |
| Promoter |  | Binding sites for heterodimers with small Maf-proteins | 0.877 | 0.776 | + | ccctGCTCactcacgcactct |
|  |  | Binding sites for heterodimers with small Maf-proteins | 0.847 | 0.776 | + | ttctGCTCtgtcatccatgcg |
| Full length gene |  | Binding sites for heterodimers with small Maf-proteins | 0.823 | 1 | - | gagtGCTGagccaaatcccca |
|  |  | Binding sites for heterodimers with small Maf-proteins | 0.831 | 0.776 | + | cccaGCTCactcagagcttgt |
|  |  | Binding sites for heterodimers with small Maf-proteins | 0.834 | 0.776 | + | cacaGCTCattcacacaccta |
|  |  | Binding sites for heterodimers with small Maf-proteins | 0.851 | 0.776 | - | acctGCTCtgtcattcaaatg |
|  |  | Antioxidant response elements | 0.83 | 1 | - | tgagggTGACtatgctcaata |
|  |  | Binding sites for heterodimers with small Maf-proteins | 0.946 | 1 | + | aggtGCTGattcacccagcct |
|  |  | Binding sites for heterodimers with small Maf-proteins | 0.872 | 0.81 | + | gataGCTTattcattgcttcc |
|  |  | NF-E2 p45 | 0.854 | 1 | - | gacctCTGActcagtttgagc |
|  |  | NF-E2 p45 | 0.869 | 1 | + | tttctCTGAttcatcactttg |
|  |  | NF-E2 p45 | 0.855 | 1 | + | taaggCTGAgccacacgacta |
|  |  | Binding sites for heterodimers with small Maf-proteins | 0.968 | 1 | + | gtgtGCTGattcatggatgta |
| ***Gstm3*** | P19639 |  |  |  |  |  |
| Promoter |  | Antioxidant response elements | 0.767 | 0.75 | - | taattgAGACacagcttcttc |
|  |  | Binding sites for heterodimers with small Maf-proteins | 0.906 | 0.81 | + | aagtGCTTactcattcattct |
|  |  | NF-E2 p45 | 0.903 | 1 | - | ttcggCTGActcaacagactc |
|  |  | NF-E2 p45 | 0.889 | 1 | - | tctatCTGActcacaagcagg |
|  |  | Binding sites for heterodimers with small Maf-proteins | 0.822 | 1 | - | tgagGCTGtctcaattccttt |
|  |  | Binding sites for heterodimers with small Maf-proteins | 0.823 | 1 | + | tgttGCTGactccaggcacac |
|  |  | NF-E2 p45 | 0.866 | 1 | + | acccgCTGAgccacctcgcca |
| Full length gene |  | NF-E2 p45 | 0.949 | 1 | + | tccagCTGActcactccatcc |
|  |  | Antioxidant response elements | 0.762 | 1 | + | tgagaaTGACttagtgcaaga |
| ***Gstm1*** | P10649 |  |  |  |  |  |
| Full length gene |  | Antioxidant response elements | 0.776 | 0.75 | - | gaaggaGGACtcagcagccct |
|  |  | NF-E2 p45 | 1 | 1 | + | atttgCTGAgtcactgggtgc |
| ***Ephx1*** | Q9D379 |  |  |  |  |  |
| Promoter |  | Binding sites for heterodimers with small Maf-proteins | 0.86 | 1 | + | ccctGCTGgttcaatgactgg |
|  |  | Binding sites for heterodimers with small Maf-proteins | 0.836 | 0.776 | + | cagtGCTCtgtcacaggagct |
|  |  | Antioxidant response elements | 0.798 | 1 | - | tcagtgTGACagtgccaaaaa |
|  |  | Antioxidant response elements | 0.79 | 0.75 | + | taagccTGGCtgagcaggagg |
|  |  | Binding sites for heterodimers with small Maf-proteins | 0.829 | 0.776 | - | catgGCTCactcactttaagg |
|  |  | Binding sites for heterodimers with small Maf-proteins | 0.826 | 1 | - | acctGCTGaaccacacacttg |
|  |  | NF-E2 p45 | 0.866 | 1 | + | aacagCTGAgccatctttcca |
|  |  | Binding sites for heterodimers with small Maf-proteins | 0.825 | 1 | + | catgGCTGcttcacaaataat |
|  |  | NF-E2 p45 | 0.877 | 0.774 | + | acatgGTGActcacaaacatc |
|  |  | NF-E2 p45 | 0.874 | 1 | - | gggcaCTGActcaggaagatc |
|  |  | Binding sites for heterodimers with small Maf-proteins | 0.842 | 1 | - | tggtGCTGactcgtaattcta |
| Full length gene |  | Binding sites for heterodimers with small Maf-proteins | 0.879 | 0.81 | + | ccctGCTTactcagggaatta |
|  |  | Binding sites for heterodimers with small Maf-proteins | 0.826 | 1 | - | cactGCTGaagcacaccagag |
|  |  | NF-E2 p45 | 0.866 | 1 | + | cacagCTGAgccaccacagca |
|  |  | Antioxidant response elements | 0.773 | 1 | - | tccttcTGACagtgcagcctg |
|  |  | NF-E2 p45 | 1 | 1 | + | atctgCTGAgtcactattagc |
|  |  | NF-E2 p45 | 0.877 | 1 | + | aactgCTGAgccatctcatca |
|  |  | Antioxidant response elements | 0.761 | 0.75 | + | tcttctTTACtaagcaaccaa |
|  |  | Antioxidant response elements | 0.767 | 0.75 | - | tcccctAGACtaagcattgag |
|  |  | Antioxidant response elements | 0.789 | 0.75 | + | acatacTGAGaaagcaggcta |
|  |  | Antioxidant response elements | 0.841 | 1 | - | acaaagTGACaaagctaaaga |
|  |  | NF-E2 p45 | 0.989 | 1 | + | atcagCTGAgtcatcggggct |
|  |  | Antioxidant response elements | 0.782 | 1 | + | tcccagTGACtttgcctgatg |
|  |  | Antioxidant response elements | 0.763 | 1 | - | taactgTGACagtgcctccag |
|  |  | NF-E2 p45 | 0.965 | 1 | + | tgatgCTGAgtcagtaagctg |
|  |  | Binding sites for heterodimers with small Maf-proteins | 0.865 | 0.776 | - | tagaGCTCagtcatctgccat |
|  |  | Antioxidant response elements | 0.767 | 1 | - | ccagccTGACccaggactgtg |
|  |  | NF-E2 p45 | 0.877 | 1 | - | aattgCTGAgccatctctcta |
|  |  | NF-E2 p45 | 0.877 | 1 | - | ccttgCTGAggcaccaggcct |
|  |  | Antioxidant response elements | 0.766 | 0.75 | + | gcctggTGCCtcagcaaggtc |
|  |  | Binding sites for heterodimers with small Maf-proteins | 0.821 | 1 | - | tctgGCTGgatcagagtggag |
|  |  | Binding sites for heterodimers with small Maf-proteins | 0.84 | 1 | - | agatGCTGagtgagacccttg |
|  |  | NF-E2 p45 | 0.989 | 1 | - | gatagCTGAgtcaccatatgg |
|  |  | Binding sites for heterodimers with small Maf-proteins | 0.825 | 0.776 | + | ggctGCTCtttcatggcaatc |
| ***Ugdh*** | O70475 |  |  |  |  |  |
| Promoter |  | Antioxidant response elements | 0.79 | 1 | + | aaagtcTGACtgggcatgctc |
|  |  | NF-E2 p45 | 0.865 | 1 | - | tccttCTGActcagcttcaca |
|  |  | NF-E2 p45 | 0.954 | 1 | + | tgaagCTGAgtcagaaggatt |
|  |  | Binding sites for heterodimers with small Maf-proteins | 0.871 | 0.776 | - | tccaGCTAagtcacgggctcc |
|  |  | Antioxidant response elements | 0.767 | 1 | + | agcccgTGACttagctggagc |
|  |  | NF-E2 p45 | 0.877 | 1 | + | aactgCTGAgccatctctcta |
|  |  | Binding sites for heterodimers with small Maf-proteins | 0.923 | 1 | - | acatGCTGtgtcattattgac |
|  |  | Antioxidant response elements | 0.789 | 0.75 | - | tttcggTGCCaaagcagaatg |
|  |  | Binding sites for heterodimers with small Maf-proteins | 0.879 | 0.776 | - | accaGCTAagtcatcctttcg |
|  |  | Binding sites for heterodimers with small Maf-proteins | 0.824 | 1 | + | gcgtGCTGagtaaactctttc |
|  |  | Binding sites for heterodimers with small Maf-proteins | 0.929 | 1 | + | ttttGCTGtgtcattccttgg |
|  |  | Binding sites for heterodimers with small Maf-proteins | 0.829 | 1 | + | acttGCTGaaccatctcacag |
| Full length gene |  | NF-E2 p45 | 0.866 | 1 | - | aatagCTGAggcatgtctcca |
|  |  | Antioxidant response elements | 0.766 | 0.75 | + | ctaataTAACtgagcatcatg |
|  |  | Binding sites for heterodimers with small Maf-proteins | 0.821 | 1 | - | ctggGCTGtatcaagatgaaa |
|  |  | NF-E2 p45 | 0.866 | 1 | - | atccgCTGAgacacccggatg |
|  |  | NF-E2 p45 | 0.866 | 1 | - | aaccgCTGAgccatctctcca |
|  |  | NF-E2 p45 | 0.889 | 1 | - | ttcatCTGActcacactacaa |
|  |  | NF-E2 p45 | 0.865 | 1 | + | atcttCTGActcagaatcctg |
